# Dance training is superior to repetitive physical exercise in inducing brain plasticity in the elderly

**DOI:** 10.1371/journal.pone.0196636

**Published:** 2018-07-11

**Authors:** Kathrin Rehfeld, Angie Lüders, Anita Hökelmann, Volkmar Lessmann, Joern Kaufmann, Tanja Brigadski, Patrick Müller, Notger G. Müller

**Affiliations:** 1 German Center for Neurodegenerative Diseases (DZNE), Magdeburg, Saxony-Anhalt, Germany; 2 Institute for Sport Science, Otto-von-Guericke University, Magdeburg, Saxony-Anhalt, Germany; 3 Institute for Physiology, Magdeburg, Saxony-Anhalt, Germany; 4 Center of Behavioral Brain Sciences (CBBS), Magdeburg, Saxony-Anhalt, Germany; 5 University Clinic for Neurology, Magdeburg, Saxony Anhalt, Germany; Vanderbilt University, UNITED STATES

## Abstract

Animal research indicates that a combination of physical activity and sensory enrichment has the largest and the only sustaining effect on adult neuroplasticity. Dancing has been suggested as a human homologue to this combined intervention as it poses demands on both physical and cognitive functions. For the present exploratory study, we designed an especially challenging dance program in which our elderly participants constantly had to learn novel and increasingly difficult choreographies. This six-month-long program was compared to conventional fitness training matched for intensity. An extensive pre/post-assessment was performed on the 38 participants (63–80 y), covering general cognition, attention, memory, postural and cardio-respiratory performance, neurotrophic factors and–most crucially–structural MRI using an exploratory analysis. For analysis of MRI data, a new method of voxel-based morphometry (VBM) designed specifically for pairwise longitudinal group comparisons was employed. Both interventions increased physical fitness to the same extent. Pronounced differences were seen in the effects on brain volumes: Dancing compared to conventional fitness activity led to larger volume increases in more brain areas, including the cingulate cortex, insula, corpus callosum and sensorimotor cortex. Only dancing was associated with an increase in plasma BDNF levels. Regarding cognition, both groups improved in attention and spatial memory, but no significant group differences emerged. The latter finding may indicate that cognitive benefits may develop later and after structural brain changes have taken place. The present results recommend our challenging dance program as an effective measure to counteract detrimental effects of aging on the brain.

## Introduction

Aging is associated with a reduction in brain volumes, mainly in the prefrontal and temporal cortices. Regions such as the occipital cortex, on the other hand, show relatively few alterations throughout the lifespan [1,2]. The changes affect both gray [2,3] and white matter [4]. The cortical volume decrease (i.e., atrophy) is not primarily caused by a loss of neurons but is due to an age-related shrinking of cells and reduced synaptic density [2,5]. In white matter, aging generates axonal deflections, resulting in defective neuronal transmission. Again, the most deflections are observed in the prefrontal cortex [6] and are associated with working memory and executive dysfunction [7].

These age-related changes, however, are not inevitable. It has become clear over the past several years that the aging brain is capable of neuroplasticity. This raises the possibility of counteracting the detrimental effects of aging on the brain. Most studies in this regard have investigated the effects of physical fitness on the aging brain. For example, Colcombe et al. (2006) reported that six months of aerobic training, in comparison to an anaerobic stretching program, led to a significant volume increase at multiple sites of the elderly subjects’ brains [8]. Others found volume and perfusion of the hippocampus, a brain region crucial for memory consolidation, to be related to the physical fitness levels of seniors [9, 10, 11]. It has been suggested that this adult neuroplasticity is mediated by BDNF, a neurotrophic factor that is also found in human blood [12, 13]. BDNF is an important growth factor supporting multiple functions in the CNS, including synaptic plasticity [14, 15, 16, 17, 18]. It has been suggested that release of BDNF from the brain is the source for circulating BDNF after physical exercise [19, 20]. An increase in circulating BDNF levels due to physical exercise has already been investigated by different groups [21] and seems to depend on exercise intensity and the type of training [22, 23, 24]. Erickson et al. (2011), for instance, demonstrate that increased hippocampal volume is associated with a greater volume of serum BDNF in seniors engaged in one year of aerobic training. From animal research, it is known that a combination of physical exercise and sensory enrichment has the strongest impact on neuroplasticity [25, 26]. Moreover, only this combination guarantees that newborn neurons survive in the long run [26]. For humans, dancing has been suggested as a homologue of this combined training, as it involves sensory, motor and cognitive challenges [27, 28]. To the best of our knowledge, only three published studies have assessed the possible impact of dancing on the brain so far [29, 30, 31]. However, these studies were cross-sectional observational studies that compared the brains of professional dancers to those of non-professionals. With such studies, there is an inevitable possibility that life-style factors other than those of interest contributed to the observed differences. Moreover, professional dancing is hardly comparable to what can be achieved in untrained healthy seniors. Therefore, in the current study, we designed a novel dance program that, on the one hand, was tailored to the needs of healthy seniors (e.g., safety) but, on the other hand, was designed to continuously challenge them by introducing new and increasingly difficult choreographies throughout the intervention. The effects of this dancing program were compared with an active, not passive, well-established sport fitness program, as we wanted to test whether our new intervention is truly superior to conventional ones. Hence, the control group engaged in sportive exercises that involved mainly repetitive exercises such as cycling. These activities were comparable regarding their cardiovascular demands but not their cognitive and coordinative demands. With this approach, we should be able to distinguish the specific effects of the dance training program from other factors, especially physical fitness. Consistent with this approach, on the brain level, we made use of a new symmetric VBM analysis method especially designed to compare longitudinal group differences.

## Materials and methods

### Subjects

**Fig 1:**
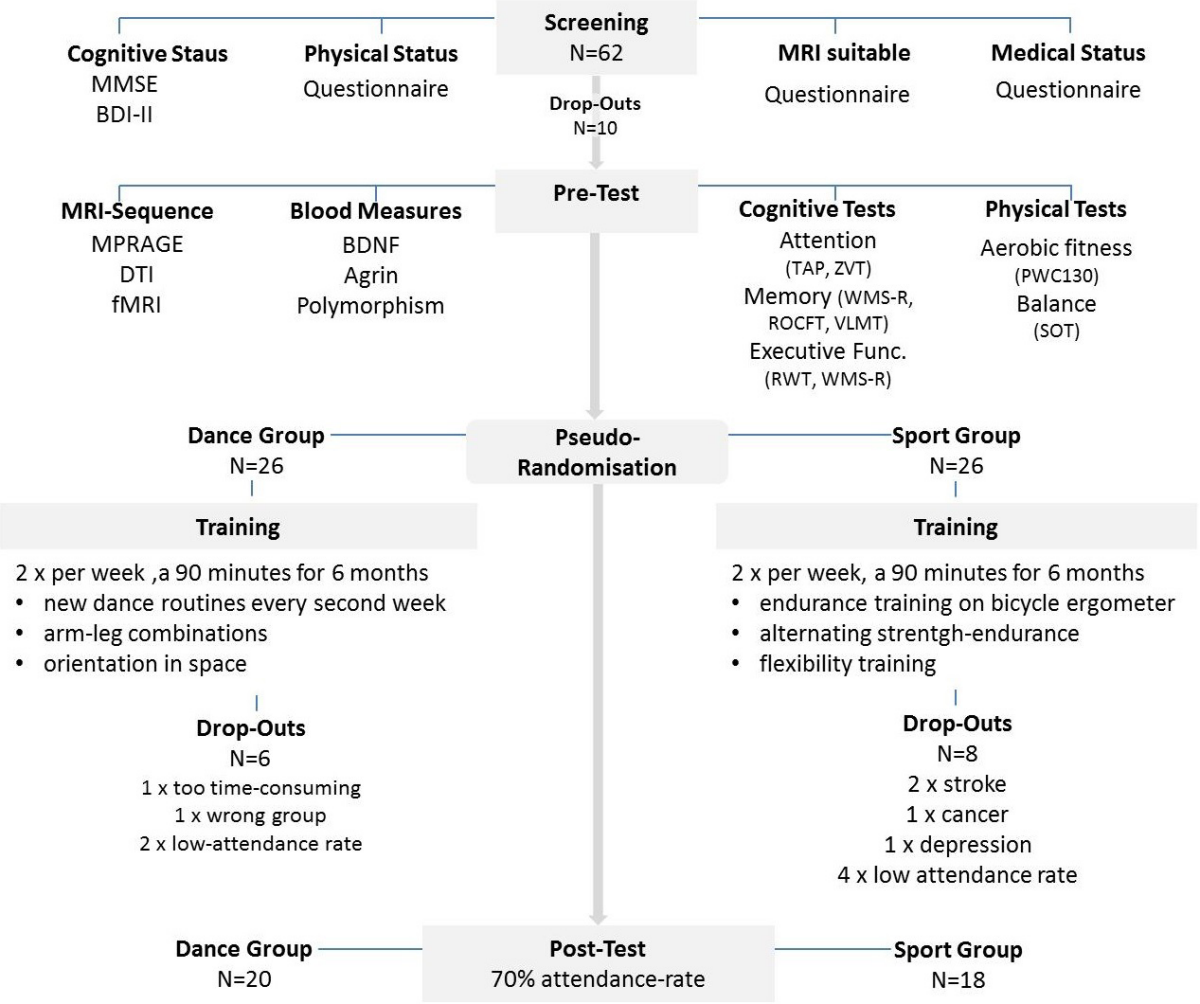
Flow chart of the study design. After all drop outs, we compared the data from 20 individuals in the experimental dance group with the data sets from 18 sportspersons in the control group.

Approval for the study was obtained from the ethics committee of the Otto-von-Guericke University, Magdeburg. All subjects signed a written informed consent and received payment for their participation. This study has been registered subsequently at DRKS (DRKS00012605), a WHO-accepted platform, in terms of omission (S1, S2 and S3 Files). The authors confirm that all ongoing and related trials for this intervention are registered. Sixty-two normal volunteers, who responded to a local advertisement, were screened. Subjects with any neurological condition, metallic implants, claustrophobia, tinnitus, BMI ≤30, high blood pressure (systolic≤140 mmHg), diabetes mellitus, intensive physical engagement (more than 1 hour/week) and abnormal performance in a cognitive screening test (MMSE < 27)[32] and a test devoted to depressive symptoms (BDI-II > 13) [33] were excluded. Fifty-two seniors (25 males; 27 females) aged 63–80 years were then randomly assigned to the experimental dance group (DG) and the control sport group (SG) controlling for age, MMSE status and physical fitness. We had some drop-outs during the training period (Fig 1), including subjects who did not reach the adherence rate of 70% (6 subjects) or got seriously sick (6 subjects) or who were unsatisfied with the group assignment (2 subjects). This left us with 38 complete data sets (S1 Table).

### Interventions

The training conditions in both groups were almost identical regarding intensity, frequency and duration. We also controlled for psychosocial effects by providing a group-based program in both study arms. Furthermore, the SG also enjoyed musical accompaniment. Both interventions occurred twice a week, lasted 90 minutes per session and were provided for six months. To control for the conditional load in both groups, we measured the heart frequency in every single training session after warm-up twice: during the main part and after cool down.

#### Dance intervention

A qualified dance instructor supervised every training session. The focus of this program was on continuous learning of new movement patterns and choreographies, thereby including coordination training and learning of specific dancing skills. Subjects were trained to accurately memorize and access different rhythms and step sequences in space, all under accuracy and time pressure. In brief, the dance intervention was divided into two blocks, each lasting three months. Each block comprised the teaching of choreographies of five different genres: line dance, jazz dance, rock ‘n’ roll, Latin-American dance and square dance. Coordinative demands and time pressure were increased in the second block by demanding more complex dance moves and introducing more beats per minute in the music.

#### Sport intervention

The SG trained according to scientific guidelines and recommendations of health sports [34] and was also supervised by a qualified trainer. Unlike the dancers, SG participants performed the same exercises repeatedly. Each session included three different units: endurance training, strength-endurance training and flexibility training. Each unit lasted 20 minutes. The endurance training was performed on bicycle ergometers with the intensity adjusted to the individual training heart frequency. The strength-endurance unit implied training with equipment such as barbells, rubber bands, Redondo balls, gymnastic sticks and fitness balls. We avoided combined arm and leg movements in order to keep coordinative demands low.

### Structural MRI scanning protocol

Magnetic resonance imaging was performed on a 3 Tesla Siemens MAGNETOM Verio scanner (Syngo MR B17). A 3-D structural MRI was acquired of each subject using a T-1-weighted MPRAGE sequence (224 sagittal slices, voxel size: 0.8 x 0.8 x 0.8 mm^3^, TR: 2500 ms, TE: 3,47 ms, TI: 1000 ms, flip angle: 7°).

#### Voxel-based morphometry for pairwise longitudinal group comparison

Since systematic bias in longitudinal image registration has recently become an issue, we used a relatively new VBM method implemented in SPM 12. This method has been developed especially for the analysis within longitudinal studies [35]. It counteracts biases from asymmetric processing, where within-subject image processing often treats one time-point differently from the others. With this new approach, it is possible to directly compare the effects of one intervention vs the other (dance vs sport) on gray and white matter structures [36].

### Analysis of BDNF

The BDNF serum and plasma levels were determined using a sandwich ELISA system (BDNF DuoSets; R&D Systems, Wiesbaden, Germany) as described previously [40, 41]. Blood samples were taken in the morning under fasting conditions.

### Cognitive assessments

We applied an extensive battery of cognitive tests that probed attention (alertness, Go/Nogo, divided attention, flexibility) [42], processing speed (trail making test) [43], verbal word fluency [44], short-term and working memory (digit span forward and backward of the Wechsler-Memory Scale) [45], verbal episodic memory (verbal learning and memory task) [46] and visuospatial memory (Rey-Osterrieth-Complex-Figure Test) [47].

### Physical fitness assessment

We employed the Physical Working Capacity 130 Test (PWC130) on a bicycle ergometer to assess endurance-related fitness. The test reflects the linear relationship between the heart rate during submaximal exercise and exercise load and highly correlates to VO_2_max [48].

### Statistical analysis

#### MRI data processing and analysis

Before preprocessing, all structural images were checked for artifacts, and the center point was placed on the anterior commissure. T1-weighted images of baseline and post measures of each subject were taken to run a pairwise longitudinal registration. The second step included the segmentation of gray and white matter as well of CSF for all average images. To measure volumetric changes in gray and white matter, every average image of each subject was disguised with the 3-D segmented maps of gray and white matter. Subsequently, spatial normalization using DARTEL (Diffeomorphic Anatomical Registration Through Exponentiated Lie Algebra) [36] was applied in the standardized MNI space. The normalized, segmented images were smoothed using an 8-mm FWHM isotropic Gaussian kernel. For each subject, normalized and smoothed images displaying volume changes in gray and white matter were created. Since we were especially interested in group differences, we investigated only average images for each individual, as in previous studies [37]. Group analyses using t-tests for independent samples compared volume changes in both gray and white matter between the two intervention groups. Co-variables such as age, sex and intracranial volume were entered in the analysis. For both groups, aligned t-contrasts (dance>sport; sport>dance) were calculated. As in previous longitudinal studies [38, 39], we applied a threshold of p< 0.001 (uncorrected) across the whole brain with a minimal cluster size of 50 voxel for gray and white matter.

### Cognitive and physical data

Explorative analysis for cognitive and physical data was performed using SPSS (SPSS 22 Inc./IBM). Intervention effects were tested using repeated-measurement ANOVAS with group (dance, sport) as the between-subject factor and time (pre, post) as the within-subject factor.

## Results

To assess baseline differences between the dance and sport groups we analyzed gray and whiter matter volume using voxel-based morphometry. Two-sample t-tests revealed no significant group differences. Baseline values of gray (p = .340) and white matter volume (p = .497) were similar for both groups and did not differ.

### Gray matter changes

**Fig 2:**
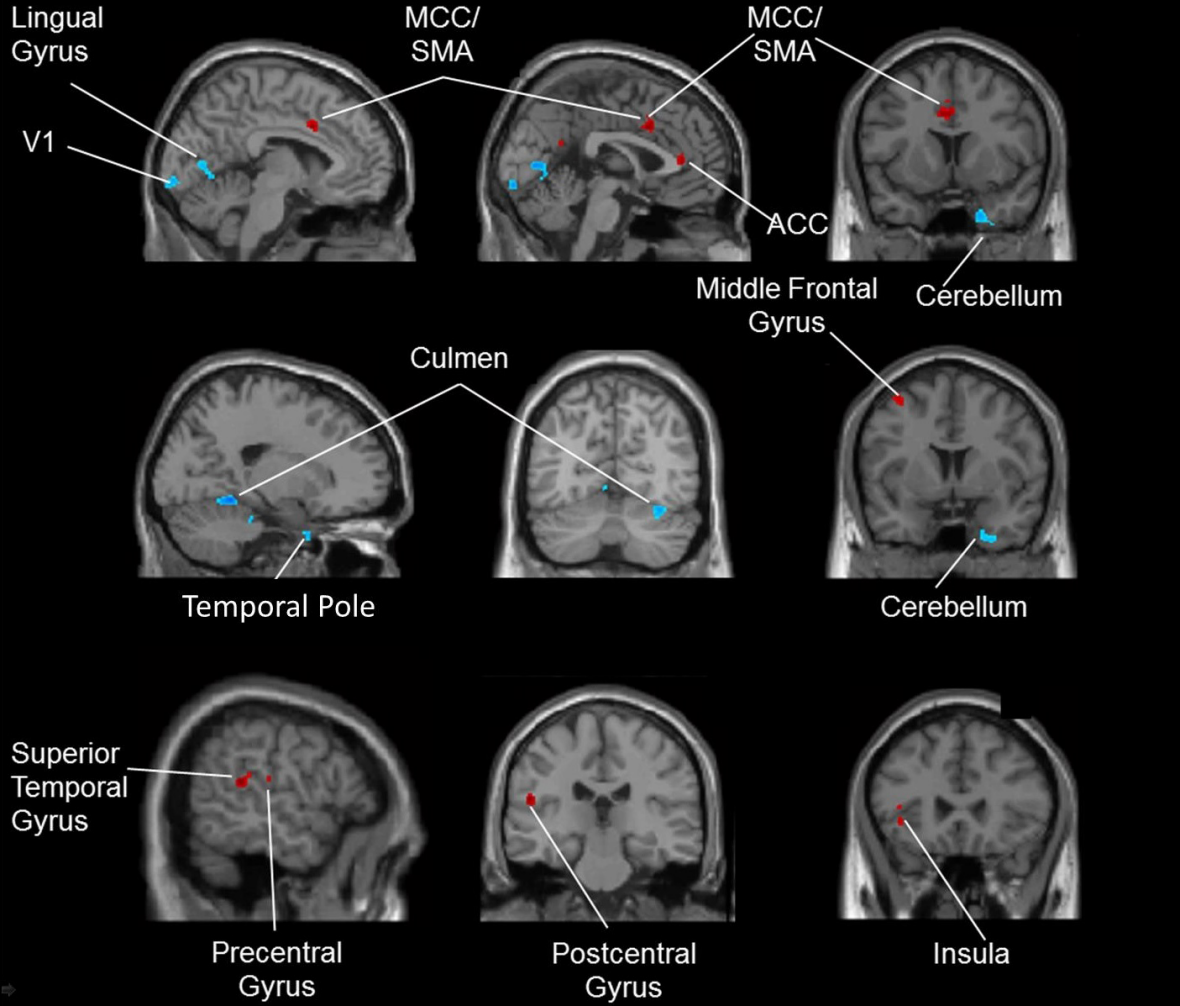
Gray matter volume increases for the contrast dance > sport (red-colored) and for the contrast sport > dance (blue-colored). *Annotation*. ACC: anterior cingulate cortex, MCC: medial cingulate cortex, SMA: supplementary motor area, V1: primary visual cortex.

After the intervention, compared to the SG, DG participants showed significantly larger volumes in multiple frontal and temporal cortical areas, including the anterior und medial cingulate cortex, the left supplementary motor area, the left precentral gyrus, the left medial frontal gyrus, the left insula, the left superior temporal gyrus and the left postcentral gyrus. The red-colored regions in Fig 2 give an overview of these brain areas. The participants from the SG showed greater volume increases compared to the dancers in occipital and cerebellar regions. These included the primary visual cortex, the left lingual gyrus, the right fusiform gyrus, the right temporal pole and the right lobe of the cerebellum. The blue-colored regions in Fig 2 give an overview of these brain regions. The corresponding MNI-coordinates and specific values for the contrast dance > sport and sport > dance are presented in S2 Table.

### White matter changes

**Fig 3:**
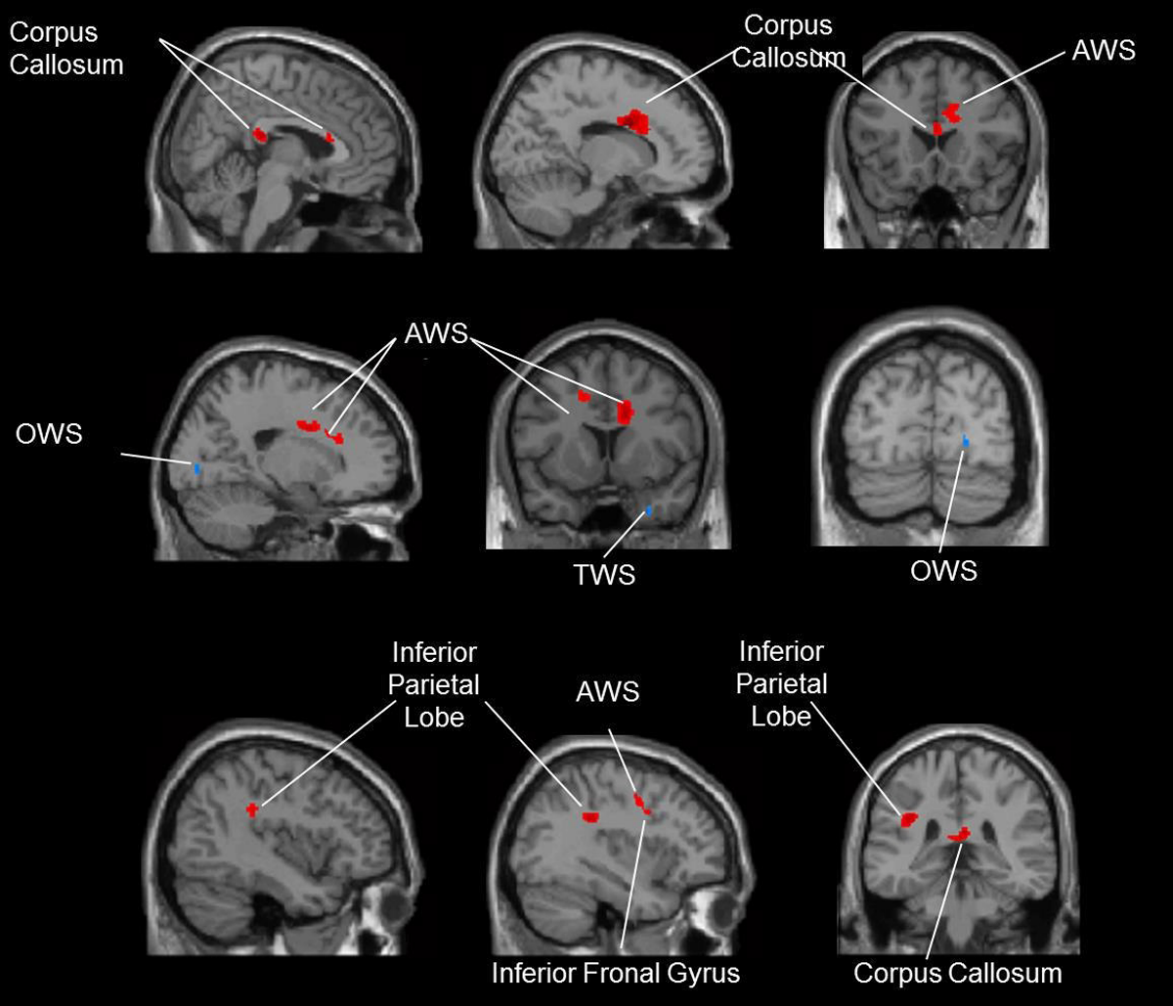
Comparison of white matter volume increases for the contrast dance > sport (red-colored) and for the contrast sport > dance (blue-colored). *Annotation*. AWS: anterior white matter, OWS: occipital white matter, TWS: temporal white matter.

Participants from the DG demonstrated larger volume increases in the truncus and splenium of the corpus callosum. An increased volume was also observed in the right and left frontal and right parietal white matter. These regions are presented in Fig 3. The SG showed greater volume increases than the dancers in the right temporal and right occipital white matter. Further information about MNI-coordinates and specific values for contrast dance > sport and sport > dance is given in S3 Table.

### Brain-derived neurotrophic factor (BDNF)

**Fig 4:**
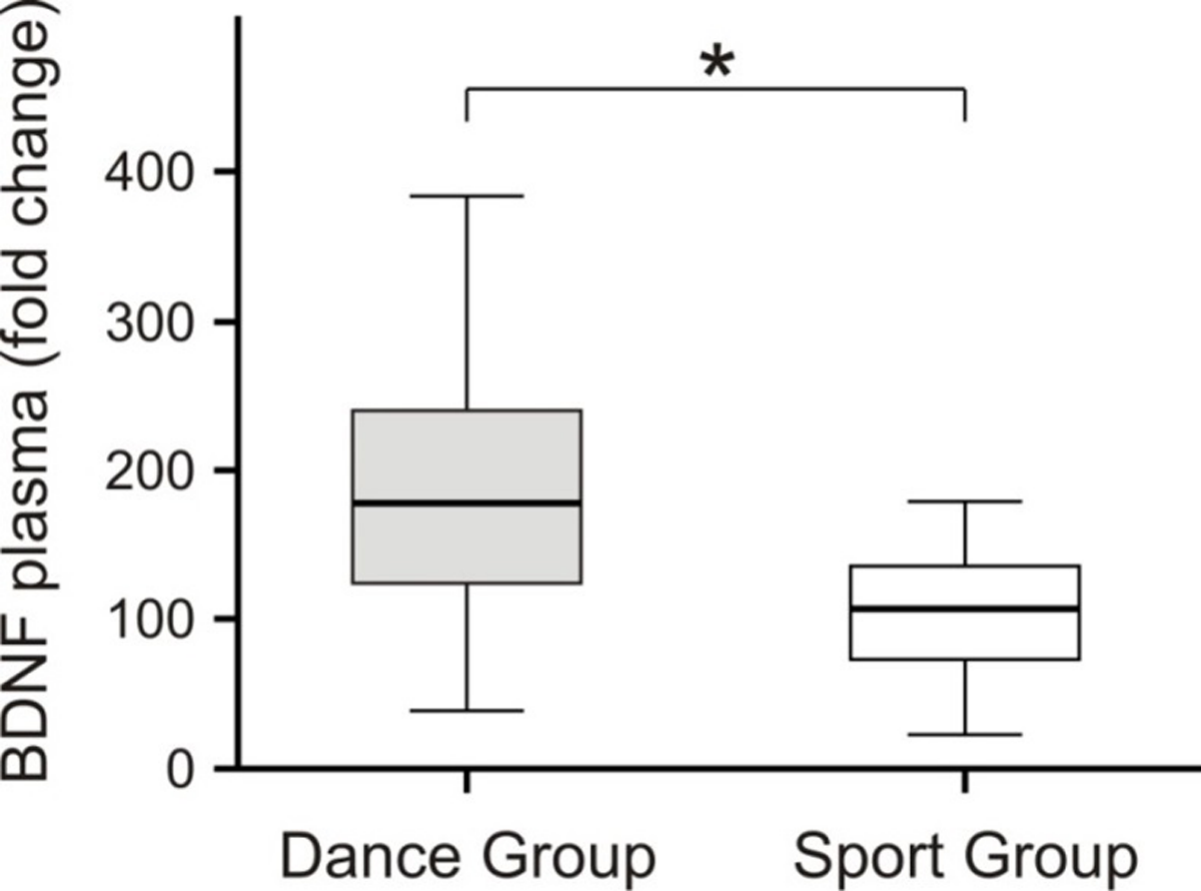
Intraindividual changes in plasma BDNF level after intervention.

Whereas no intervention effects were observed in BDNF serum values, the plasma values showed an increase after dance training. This is confirmed by a time x group interaction with F(1,35) = 4,3; p = 0.046; η^2^ = ,115. Although the dance group started with a lower plasma BDNF level, a t-test did not reveal a significant between-group difference at baseline (S4 Table). To exclude interindividual variations, we calculated intraindividual changes in BDNF levels after intervention. Again, DG participants showed significantly larger increases in plasma BDNF level than SG participants (Fig 4).

As it is evident from the figure, the increase in BDNF level was significantly increased in the DG compared with the SG (Mann-Whitney’s U-test, p < 0.018).

### Cognitive results

We found a main effect of time for alertness with acoustic cues (F (1,37) = 9,48; p = .004; η^2^ = ,218) but no time x group interaction. Visuospatial memory improved after both trainings, demonstrated by a main effect of time for immediate (F(1,37) = 38,04; p < .001; η^2^ = ,54) as well as for delayed recall (F(1,37) = 28,37; p < .001; η^2^ = ,455). There was, however, no significant group x time interaction (all p-values > 0.6). Other cognitive functions did not change in either group (all p-values > 0.28). For detailed information see S5, S6 and S7 Tables.

### Physical fitness results

After six months of training, both groups enhanced their aerobic fitness, indicated by a significant main effect of time for the PWC 130 test (F(1,32) = 11,4; p = 0.002; η^2^ = ,275). No group differences emerged.

## Discussion

This study examined the effects of a specially designed dance training program requiring constant learning of new choreographies compared to a conventional sportive fitness training with mainly repetitive exercises on brain structure and function in healthy seniors. We used a new voxel-based morphometry approach specifically designed for a pairwise longitudinal group comparison. Both groups improved their fitness levels. However, as there was no group difference, the observed differences in brain plasticity cannot simply be attributed to physical fitness but must reflect the specific components of the intervention programs.

### Gray matter changes in dancers

In general, dancing induced more wide-spread gray matter increases than the sportive control program. The dancers showed gray matter volume increases after training in the anterior and medial cingulate cortex, in the left supplementary motor area, in the left sided precentral gyrus, in the left medial frontal gyrus, in the left superior temporal gyrus, in the left insula and in the left post-central gyrus.

The anterior cingulate cortex and the medial frontal gyri are mainly associated with working memory [49, 50]. These areas also form the basis of executive functions, cognitive control and attention regulation. In addition, the cingulate gyrus is also a part of the limbic system and is engaged in the storage of new information in long-term-memory, as well as in the retrieval of stored information [50]. In the insula, the networks of working memory and attention overlap and potentially interact [51, 52]. Dancing poses demands on both attention and memory. Our dancing program was intended to keep the dancers in a constant learning process by confronting them continuously with novel motion sequences. They had to attentively follow the movements of the instructor to retain, retrieve and perform the choreographies under several conditions of pressure. Since the genre or the dancing style was changed every second week, no monotony could evolve. Rather this approach placed high demands on attentional and memory processes, especially working memory [53]. These cognitive functions usually underlie an age-related degradation making the results especially promising with respect to a preventive potential.

Enhanced volumes after dance training were also seen in motor and somatosensory areas. Among these were the precentral gyri and the supplementary motor area. These areas have a preprocessing and executive function within the motor system [54, 55]. Again, the increased volumes of these regions can be attributed to the complexity of the dance movement patterns. The simultaneous use of different body parts for different rhythms promotes the coordination of the whole body. In addition, the changing tempi of the music induce a dynamic and temporal variability in the execution of partial (segmented) and whole-body movements. Dancing also increased the volume of the postcentral gyrus. In this area, somatosensory fibers end, which convey information from proprioceptive organs such as neuromuscular spindles, joint and sinew receptors. Other than the fitness program, our dancing program involved manifold steps and constant shifts of balance and regular turns. Hence, it can be assumed that body movement perception was trained as well as the perception of the location and position of the body and body segments to each other in space. This kind of feedback (reafference) to the cortex is needed to align and refine movement processes.

Additionally, the superior temporal gyrus displayed an increased gray matter volume after six months of dance training. This part of the temporal lobe is associated with episodic memory and deteriorates early in Alzheimer’s disease [8]. Furthermore, it is closely linked to the adjacent superior temporal sulcus, which is crucial for auditory short-term memory [56] and audio-visual integration [57, 58]. While learning to dance, participants needed to keep the verbal instructions of the teacher in mind. Another important aspect constitutes the audio-visual integration of stimuli because dancing involves multisensory stimulation with visual, sensory and auditory input. For example, dancers have to visually observe and sense their movements while they listen to music and have to set these pieces of information in accordance with each other.

### White matter changes in dancers

White matter volume increases associated with dancing were seen in close spatial proximity to the cortical changes in frontal and parietal parts of the brains. Most outstanding was the enlargement of the corpus callosum, which carries the largest part of the commissure fibers and connects nearly all parts of the hemispheres. Hence, the corpus callosum ensures the communication between both cerebral hemispheres. Aging leads to degradation of this region, a process that has been associated with age-related loss in cognitive performance [59]. The present findings indicate that dancing can intensify the connectivity and interaction between both cerebral hemispheres. This appears plausible given that different motor, somatosensory and cognitive areas have to communicate in order to accomplish the complex task of dancing.

### Brain changes after sport training

Compared to dancing, the completion of the sport program led to fewer and smaller brain volume increases. For both white and gray matter, they were seen mainly in the cerebellum and higher visual and adjacent areas. The increased volume in the cerebellum seems plausible. Compared to dancing, the movements of the sport group had a cyclic and repetitive character. After the initial phase of training, these routines hardly required any conscious control; instead, they were performed automatically [28]. The cerebellum is crucial for unconscious planning and execution of movements [60]. Furthermore, together with the basal ganglia, it constitutes the basis of procedural memory [61, 62]. In procedural memory, the retrieval of movement programs does not underlie conscious cognitive control and does not require focused attention. In sum, in contrast to dancing, the fitness training required less cognitive resources.

The volume change in visual areas including the right fusiform gyrus came rather unexpectedly. Initially, it is unclear why fitness training should boost areas related to visual perception, especially that of faces, more than dancing [63]. However, as outlined above, dancing stresses audio-visual integration rather than visual perception per se. The fitness training, on the other hand, with its low cognitive demands, may have seduced participants to focus their attention on visual input–especially the faces of the other participants.

### Possible neurophysiological basis of the observed volume changes

Animal models have suggested that age limits the capacity for adaptive changes related to dynamic neural network modulations induced by environmental and intrinsic changes [64]. On the other hand, it is known that new neurons are generated in the vertebrate brain throughout the life span [65]. This neurogenesis increases with physical exercise and occurs in the hippocampus as well as in the subventricular zone around the lateral ventricles [66]. However, a large fraction of the new neurons does not survive [26]. It was stated that while exercise increases the rate of neurogenesis, environmental enrichment increases the ratio of new neurons that survive and get incorporated into the lattice of the existing neural network [67]. In this respect, dancing, which can be considered a combination of exercise and sensory enrichment, may have a similar potential for inducing long-lasting survival of newly created neurons. BDNF may be a possible mediating factor of adult neuroplasticity [13]. In the present study, BDNF plasma levels increased during the dance training. BDNF is known to be important for synaptogenesis, dendritogenesis as well as neurogenesis [14, 15, 16, 17, 18]. Activity-dependent release of BDNF during dancing, a training program that involves attention and memory process could underlie the observed volume increases of gray matter. In Alzheimer’s disease, known for its neurodegenerative process and cognitive decline, BDNF levels in the brain and also in the blood were found to be significantly reduced [68]. Therefore, the observed increase in gray matter volume associated with the increase in circulating BDNF support the idea of BDNF-induced structural changes in the brain.

### Cognitive effects

Compared with the effects on the brain, the effects of the trainings on cognitive functions were rather low and showed no significant differences between the groups. Improvements of some attentional processes and of visual spatial memory were observed in both groups. This raises the general question of why the effects on cognition were so small compared to those induced in the brain. Two explanations will be discussed: First, the tests were not sensitive for revealing changes in cognitive processes. Second, the brain changes may precede the changes in measurable behavior, so that the latter would have required a longer intervention program. This assumption is currently under investigation in our laboratory.

### Limitations

Like other studies on training induced neuroplasticity [38, 39], our MRI results are based on uncorrected data because with the small sample size, the results would not have survived traditional corrections such as FDR. Next, we tested highly selected, healthy and motivated samples of elder adults; hence, whether the results generalize to the elderly population as a whole needs to be investigated in future studies. Lastly, the assessment of subcortical structures with VBM has been criticized as being less reliable [69]. Future studies should make use of DTI white matter tractography to assess white matter changes.

### Conclusions

We were able to demonstrate in a randomized intervention trial that dancing has a strong potential to induce more positive effects on brain volumes in elderly people. Previous research has mainly focused on other, more monotonic aerobic exercises such as running, walking, cycling (endurance training) or on anaerobic strength and stretching exercises. Compared to these standard fitness programs, our specially designed six-month dancing program increased volumes in regions which relate to higher cognitive processes such as working memory and attention and which are especially affected by age-related decline. In our view, the more pronounced effects of dancing on the human brain can be explained by the fact that dancing promotes a large number of processes at the same time: spatial orientation, movement coordination, balance, endurance, interaction and communication. Furthermore, by presenting our participants with ever new choreographies, our program induced a constant learning processes. This dance training program does not need special requirements, has a low risk of accidents and can be implemented at low costs. Therefore, it endorses itself as an appropriate measure to counteract age-related declines in brain structure.

## Supporting information

S1 Table Characteristics of the dance and sport group (M = Mean; SD = Standard deviation). *Annotation*. BMI = Body-Mass-Index; BDI-II = Becks-Depressions-Inventar II; MMSE = Mini Mental State Examination.
(PDF)

S2 Table MNI-coordinates and statistical values for gray matter. *Annotation*. lGTS = left gyrus temporalis superior, lGPre = left gyrus precentralis, MCC = medial cingular cortex, lSMA = left supplementary-motor area, lGPo = left gyrus postcentralis, ACC = anterior cingular cortex, lGFM = left gyrus frontalis medius, rGF = right gyrus fusiformis, lGL = left gyrus lingualis, rTP = right temporalpol, V1 = primary visual cortex, ***p ≤ .001 (uncorrected).
(PDF)

S3 Table MNI-coordinates and statistical values for white matter. *Annotation*. CC-T = truncus of corpus callosum, CC-S = splenium of corpus callosum, AWS = anterior white matter, PWS = posterior white matter, TWS = temporal white matter, OWS = occipital white matter, ***p ≤ .001 (uncorrected).
(PDF)

S4 Table BDNF serum and plasma levels before and after intervention. *Annotation*. Repeated-measures ANOVA following post hoc pairwise comparison (Bonferroni).
(PDF)

S5 Table Mean and standard deviation of performances in the domain *attention* in both groups. *Annotation*. Testbattery for Attention: Alter_vis = alertness (visual), Alert_aud = alertness (+ auditive distractor), Go/NoGo = selective attention (RT = reaction time; F = false; M = missed), D_Att_vis = devided attention (visual), Dev_Att_aud = devided attention (auditive), Flex = flexibility; ZVT = Zahlenverbindungstest (trail making; processing speed), level of significance: p < .05.
(PDF)

S6 Table Mean and standard deviation of performances in the domain *memory* in both groups. *Annotation*. VLMT = Verbaler Lern-und Merkfähigkeitstest (verbal learning and memory test): VLMT-L = Learning, VMLT_RI = recall after interference list, VLMT_DR = delayed recall, VLMT_REC = recognition; ROCFT = Rey-Osterrieth Complex Figure Test, ROCFT_C = copy, ROCFT_IR = immeadiate recall, ROCFT_DR = delayed recall, ROCFT_REC = recognition; WMS_DS_fw = Wechselr-Memory Scale (Digit Span forward); pt = points.
(PDF)

S7 Table Mean and standard deviation of performances in the domain *executive functions* in both groups. *Annotation*. WMS_DS_bw = Wechsler Memory Scale (Digit Span backward); RWT = Regensburger Wortflüssigkeitstest (verbal word fluency), RWT_M-Words = formallexikal word fluency, RWT_G-R-words = formallexikal. shift between categories, RWT_animals = semantic word fluency, RWT_Cl-Fl = Clothing and flowers (semantic shift of category).
(PDF)

S1 File TREND Checklist. (PDF)

S2 File Study protocol (DRKS00012605). (PDF)

S3 File Study protocol approved by ethical commitee. (DOCX)

## References

[1] Hedden T, Gabrieli J-D (2004) Insights into the ageing mind: a view from cognitive neuroscience. *Nature reviews neuroscience* 5(2): 87–96. doi: 10.1038/nrn1323 14735112 10.1038/nrn1323

[2] Resnick S-M, Pham D-L, Kraut M-A, Zonderman A-B, Davatzikos C (2003) Longitudinal magnetic resonance imaging studies of older adults: a shrinking brain. *Journal of Neu-roscience* 23(8): 3295–3301. 10.1523/JNEUROSCI.23-08-03295.2003 PMC6742337 12716936

[3] Haug H, Eggers R (1991) Morphometry of the human cortex cerebri and corpus striatum during aging. *Neurobiology of aging* 12(4): 336–338. 1961364 10.1016/0197-4580(91)90013-a

[4] Gunning‐Dixon F-M, Brickman A-M, Cheng J-C, Alexopoulos G-S (2009) Aging of cerebral white matter: a review of MRI findings. *International journal of geriatric psychiatry* 24(2): 109–117. doi: 10.1002/gps.2087 18637641 10.1002/gps.2087 PMC2631089

[5] Terry R-D, Katzman R (2001) Life span and synapses: will there be a primary senile dementia? *Neurobiology of Aging* 22(3): 347–348. 11378236 10.1016/s0197-4580(00)00250-5

[6] Buckner R-L (2004) Memory and executive function in aging and AD: multiple factors that cause decline and reserve factors that compensate. *Neuron* 44(1): 195–208. doi: 10.1016/j.neuron.2004.09.006 15450170 10.1016/j.neuron.2004.09.006

[7] Berchtold N-C, Cotman C-W (2009) Normal and pathological aging: from animals to humans. *In Animal Models of Human Cognitive Aging* (Humana Press), pp 1–28.

[8] Colcombe S-J, Erickson K-I, Scalf P-E, Kim J-S, Prakash R, McAuley E, et al (2006) Aerobic exercise training increases brain volume in aging humans. The Journals of Gerontology Series A: Biological Sciences and Medical Sciences 61(11): 1166–1170. 10.1093/gerona/61.11.1166 17167157

[9] Erickson K-I, Prakash R-S, Voss M-W, Chaddock L, Hu L, Morris K-S, et al (2009) Aerobic fitness is associated with hippocampal volume in elderly humans. *Hippocampus* 19(10): 1030–1039. doi: 10.1002/hipo.20547 19123237 10.1002/hipo.20547 PMC3072565

[10] Erickson K-I, Voss M-W, Prakash R-S, Basak C, Szabo A, Chaddock L, et al (2011) Exercise training increases size of hippocampus and improves memory. *Proceedings of the National Academy of Sciences* 108(7): 3017–3022. 10.1073/pnas.1015950108 PMC3041121 21282661

[11] Maass A, Düzel S, Goerke M, Becke A, Sobieray U, Neumann K, et al (2015). Vascular hippocampal plasticity after aerobic exercise in older adults. *Molecular psychiatry* 20(5): 585–593. doi: 10.1038/mp.2014.114 25311366 10.1038/mp.2014.114

[12] Pereira A-C, Huddleston D-E, Brickman A-M, Sosunov A-A, Hen R, McKhann G-M, et al (2007) An in vivo correlate of exercise-induced neurogenesis in the adult dentate gyrus. *Proceedings of the National Academy of Sciences* 104(13): 5638–5643. 10.1073/pnas.0611721104 PMC1838482 17374720

[13] Flöel A, Ruscheweyh R, Krüger K, Willemer C, Winter B, Völker K, et al (2010) Physical activity and memory functions: are neurotrophins and cerebral gray matter volume the missing link?. *Neuroimage* 49(3): 2756–2763. doi: 10.1016/j.neuroimage.2009.10.043 19853041 10.1016/j.neuroimage.2009.10.043

[14] Edelmann E, Leßmann V, Brigadski T (2014) Pre-and postsynaptic twists in BDNF secretion and action in synaptic plasticity. *Neuropharmacology* 76: 610–627. doi: 10.1016/j.neuropharm.2013.05.043 23791959 10.1016/j.neuropharm.2013.05.043

[15] Huang E-J, Reichardt L-F (2001) Neurotrophins: roles in neuronal development and function. *Annual review of neuroscience* 24: 677–736. doi: 10.1146/annurev.neuro.24.1.677 11520916 10.1146/annurev.neuro.24.1.677 PMC2758233

[16] Klein R (1994) Role of neurotrophins in mouse neuronal development. *The FASEB Journal* 8(10): 738–744. 8050673 10.1096/fasebj.8.10.8050673

[17] Leßmann V, Brigadski T (2009) Mechanisms, locations, and kinetics of synaptic BDNF secretion: an update. *Neuroscience research* 65(1): 11–22. doi: 10.1016/j.neures.2009.06.004 19523993 10.1016/j.neures.2009.06.004

[18] Park H, Poo M-M (2013) Neurotrophin regulation of neural circuit development and function. *Nature Reviews Neuroscience* 14(1): 7–23. doi: 10.1038/nrn3379 23254191 10.1038/nrn3379

[19] Brigadski T, Leßmann V (2014) BDNF: a regulator of learning and memory processes with clinical potential. *e-Neuroforum* 5(1): 1–11.

[20] Rasmussen P, Brassard P, Adser H, Pedersen M-V, Leick L, Hart E, et al (2009). Evidence for a release of brain‐derived neurotrophic factor from the brain during exercise. *Experimental physiology* 94(10): 1062–1069. doi: 10.1113/expphysiol.2009.048512 19666694 10.1113/expphysiol.2009.048512

[21] Huang T, Larsen K-T, Ried‐Larsen M, Møller N-C, Andersen L-B (2014) The effects of physical activity and exercise on brain‐derived neurotrophic factor in healthy humans: A review. *Scandinavian journal of medicine & science in sports* 24(1): 1–10. 23600729 10.1111/sms.12069

[22] Vega S-R, Strüder H-K, Wahrmann B-V, Schmidt A, Bloch W, Hollmann W (2006) Acute BDNF and cortisol response to low intensity exercise and following ramp incremental exercise to exhaustion in humans. *Brain research* 1121(1): 59–65. doi: 10.1016/j.brainres.2006.08.105 17010953 10.1016/j.brainres.2006.08.105

[23] Zoladz J-A, Pilc A, Majerczak J, Grandys M, Zapart-Bukowska J, Duda K (2008) Endurance training increases plasma brain-derived neurotrophic factor concentration in young healthy men. *J Physiol Pharmacol* 59: 119–132. 19258661

[24] Seifert T, Brassard P, Wissenberg M, Rasmussen P, Nordby P, Stallknecht B, et al (2010) Endurance training enhances BDNF release from the human brain. *American Journal of Physiology-Regulatory*, *Integrative and Comparative Physiology* 298(2): R372–R377. 10.1152/ajpregu.00525.2009 19923361

[25] Van Praag H, Shubert T, Zhao C, Gage F-H (2005) Exercise enhances learning and hippocampal neurogenesis in aged mice. *The Journal of neuroscience* 25(38): 8680–8685. doi: 10.1523/JNEUROSCI.1731-05.2005 16177036 10.1523/JNEUROSCI.1731-05.2005 PMC1360197

[26] Kempermann G, Fabel K, Ehninger D, Babu H, Leal-Galicia P, Garthe A, et al (2010) Why and how physical activity promotes experience-induced brain plasticity. *Frontiers in neuroscience* 4(189): 1–9. 21151782 10.3389/fnins.2010.00189 PMC3000002

[27] Kattenstroth J-C, Kolankowska I, Kalisch T, Dinse H-R (2010) Superior sensory, motor, and cognitive performance in elderly individuals with multi-year dancing activities. *Frontiers in Aging Neuroscience* 2(31): 1–9. 20725636 10.3389/fnagi.2010.00031 PMC2917240

[28] Rehfeld K, Hökelmann A, Lehmann W, Blaser P (2014) Auswirkungen einer Tanz- und Kraft-Ausdauer-Intervention auf kognitive Fähigkeiten älterer Menschen. *Zeitschrift für Neuropsychologie* 25(2): 99–108.

[29] Brown S, Martinez M-J, Parsons L-M (2006) The neural basis of human dance. *Cerebral cortex* 16(8): 1157–1167. doi: 10.1093/cercor/bhj057 16221923 10.1093/cercor/bhj057

[30] Hänggi J, Koeneke S, Bezzola L, Jäncke L (2010) Structural neuroplasticity in the sensorimotor network of professional female ballet dancers. *Human brain mapping* 31(8): 1196–1206. doi: 10.1002/hbm.20928 20024944 10.1002/hbm.20928 PMC6870845

[31] Hüfner K, Binetti C, Hamilton D-A, Stephan T, Flanagin V-L, Linn J, et al (2011) Structural and functional plasticity of the hippocampal formation in professional dancers and slackliners. *Hippocampus* 21(8): 855–865. doi: 10.1002/hipo.20801 20572197 10.1002/hipo.20801

[32] Folstein M-F, Folstein S-E, McHugh P-R (1975) “Mini-mental” state. A practical method for grading the state of patients for the clinician. *Journal of Psychiatric Research* 12(3): 189–198. 1202204 10.1016/0022-3956(75)90026-6

[33] Beck A-T, Brown G-K, Steer R-A (2006) *Beck-Depressions-Inventar* BDI-II, Manual. 2. Auflage (Harcourt Test Services, Frankfurt/M).

[34] Brehm W, Janke A, Sygusch R, Wagner P (2006) *Gesund durch Gesundheitssport* (Juventa, München), pp 16–23.

[35] Ashburner J, Ridgway G-R (2013) Symmetric diffeomorphic modeling of longitudinal structural MRI. *Frontiers in neuroscience* 6 (197): 1–19. 10.3389/fnins.2012.00197 PMC3564017 23386806

[36] Ashburner J (2007) A fast diffeomorphic image registration algorithm. *Neuroimage* 38(1): 95–113. doi: 10.1016/j.neuroimage.2007.07.007 17761438 10.1016/j.neuroimage.2007.07.007

[37] Melzer T-R, Myall D-J, MacAskill M-R, Pitcher T-L, Livingston L, Watts R, et al (2015) Tracking Parkinson’s Disease over One Year with Multimodal Magnetic Resonance Imaging in a Group of Older Patients with Moderate Disease. *PloS one* 10(12): 1–14. 10.1371/journal.pone.0143923 PMC4694717 26714266

[38] Boyke J, Driemeyer J, Gaser C, Büchel C, May A (2008) Training-induced brain structure changes in the elderly. *The Journal of neuroscience* 28(28): 7031–7035. doi: 10.1523/JNEUROSCI.0742-08.2008 18614670 10.1523/JNEUROSCI.0742-08.2008 PMC6670504

[39] Draganski B, Gaser C, Busch V, Schuierer G, Bogdahn U, May A (2004) Neuroplasticity: changes in grey matter induced by training. *Nature* 427(6972): 311–312. doi: 10.1038/427311a 14737157 10.1038/427311a

[40] Schega L, Peter B, Brigadski T, Leßmann V, Isermann B, Hamacher D, et al (2016) Effect of intermittent normobaric hypoxia on aerobic capacity and cognitive function in older people. *Journal of Science and Medicine in Sport*. 10.1016/j.jsams.2016.02.012 27134133

[41] Petzold A, Psotta L, Brigadski T, Endres T, Lessmann V (2015) Chronic BDNF deficiency leads to an age-dependent impairment in spatial learning. *Neurobiology of learning and memory* 120: 52–60. doi: 10.1016/j.nlm.2015.02.009 25724412 10.1016/j.nlm.2015.02.009

[42] Zimmermann P, Fimm B (2002) *Testbatterie zur Aufmerksamkeitsprüfung* (TAP), Version 1.7, Handbuch (Psytest, Würselen).

[43] Oswald W-D, Roth E (1978) *Der Zahlen-Verbindungs-Test (ZVT)*, Ein sprachfreier Intelligenz-Test zur Messung der “kognitiven Leistungsgeschwindigkeit”, Handanweisung, 2. Auflage (Hogrefe Verlag für Psychologie, Göttingen).

[44] Aschenbrenner S, Tucha O, Lange K-W (2000) *Manual zum RWT (Regensburger Wortflüssigkeits-Test)*, Handanweisung (Hogrefe Verlag, Göttingen).

[45] Härting C, Markowitsch H-J, Neufeld H, Calabrese P, Deisinger K, Kessler J (2000) *Wechsler Gedächtnis Test-Revidierte Fassung* (WMS-R) (Huber, Bern).

[46] Helmstaedter C, Lendt M, Lux S (2001) *Verbaler Lern- und Merkfähigkeitstest*, VLMT, Manual (Beltz Test, Göttingen).

[47] Meyers J-E, Meyers K-R (1995) *Rey Complex Figure Test and Recognition Trial*. (Psychological Assessment Resources, Florida).

[48] Stemper T (2003) *Lehrbuch Lizenzierter Fitness-Trainer DSSV* (SSV-Verlag, Hamburg).

[49] McCarthy G, Puce A, Constable R-T, Krystal J-H, Gore J-C, Goldman-Rakic P (1996) Activation of human prefrontal cortex during spatial and nonspatial working memory tasks measured by functional MRI. *Cerebral Cortex* 6(4): 600–611. 8670685 10.1093/cercor/6.4.600

[50] Kukolja J, Fink G-R (2011) Amnesie. *Neurologische Differenzialdiagnostik*. *Evidenzbasierte Entscheidungsprozesse und diagnostische Pfade*, eds Bewermeyer H, Fink G-R, Limmroth V (Schattauer, Stuttgart), pp 41–50.

[51] Mayer J-S, Bittner R-A, Nikolić D, Bledowski C, Goebel R, Linden D-E-J (2007) Common neural substrates for visual working memory and attention. *Neuroimage* 36(2): 441–453. doi: 10.1016/j.neuroimage.2007.03.007 17462914 10.1016/j.neuroimage.2007.03.007

[52] Soros P, Marmurek J, Tam F, Baker N, Staines W-R, Graham S-J (2007) Functional MRI of working memory and selective attention in vibrotactile frequency discrimination. BMC. *Neuroscience* 8(1): 48. 17610721 10.1186/1471-2202-8-48 PMC1925104

[53] Kattenstroth J-C, Kalisch T, Holt S, Tegenthoff M, Dinse H-R (2013) Six months of dance intervention enhances postural, sensorimotor, and cognitive performance in elderly without affecting cardio-respiratory functions. *Frontiers in aging neuroscience* 5(5): 1–9. 23447455 10.3389/fnagi.2013.00005 PMC3581819

[54] Picard N, Strick P-L (2001) Imaging the premotor areas. *Current opinion in neurobiology* 11(6): 663–672. 11741015 10.1016/s0959-4388(01)00266-5

[55] Ball T, Schreiber A, Feige B, Wagner M, Lücking C-H, Kristeva-Feige R (1999) The role of higher-order motor areas in voluntary movement as revealed by high-resolution EEG and fMRI. *Neuroimage* 10(6): 682–694. doi: 10.1006/nimg.1999.0507 10600414 10.1006/nimg.1999.0507

[56] Leff A-P, Schofield T-M, Crinion J-T, Seghier M-L, Grogan A, Green D-W, et al (2009) The left superior temporal gyrus is a shared substrate for auditory short-term memory and speech comprehension: evidence from 210 patients with stroke. *Brain* 132(12): 3401–3410. 19892765 10.1093/brain/awp273 PMC2792373

[57] Stevenson R-A, James T-W (2008) Audiovisual integration in human superior temporal sulcus: Inverse effectiveness and the neural processing of speech and object recognition. *Neuroimage* 44(3): 1210–1223. doi: 10.1016/j.neuroimage.2008.09.034 18973818 10.1016/j.neuroimage.2008.09.034

[58] Willems R-M, Özyürek A, Hagoort P (2009) Differential roles for left inferior frontal and superior temporal cortex in multimodal integration of action and language. *NeuroImage*, 47(4): 1992–2004. doi: 10.1016/j.neuroimage.2009.05.066 19497376 10.1016/j.neuroimage.2009.05.066

[59] O’Sullivan M, Jones D-K, Summers P-E, Morris R-G, Williams S-C-R, Markus H-S (2001) Evidence for cortical “disconnection” as a mechanism of age-related cognitive decline. *Neurology* 57(4): 632–638. 11524471 10.1212/wnl.57.4.632

[60] Thach W-T (1998) A role for the cerebellum in learning movement coordination. *Neurobiology of learning and memory* 70(1): 177–188. 9753595 10.1006/nlme.1998.3846

[61] Doyon J, Penhune V, Ungerleider L-G (2003) Distinct contribution of the cortico-striatal and cortico-cerebellar systems to motor skill learning. *Neuropsychologia* 41(3): 252–262. 12457751 10.1016/s0028-3932(02)00158-6

[62] Haber S-N, Calzavara R (2009) The cortico-basal ganglia integrative network: the role of the thalamus. *Brain research bulletin* 78(2): 69–74. 18950692 10.1016/j.brainresbull.2008.09.013 PMC4459637

[63] Kanwisher N, McDermott J, Chun M-M (1997) The fusiform face area: a module in human extrastriate cortex specialized for face perception. *The Journal of neuroscience* 17(11): 4302–4311. 9151747 10.1523/JNEUROSCI.17-11-04302.1997 PMC6573547

[64] Wagner A-P, Schmoll H, Badan I, Platt D, Kessler C (2000) Brain plasticity: to what extent do aged animals retain the capacity to coordinate gene activity in response to acute challenges. *Experimental gerontology* 35(9): 1211–1227. 11113603 10.1016/s0531-5565(00)00154-6

[65] Colucci‐D'Amato L, di Porzio U (2008) Neurogenesis in adult CNS: from denial to opportunities and challenges for therapy. *Bioessays*: 30(2): 135–145. doi: 10.1002/bies.20703 18200551 10.1002/bies.20703

[66] Van Praag H, Kempermann G, Gage F-H (1999) Running increases cell proliferation and neurogenesis in the adult mouse dentate gyrus. *Nature neuroscience* 2(3): 266–270. doi: 10.1038/6368 10195220 10.1038/6368

[67] Kronenberg G, Reuter K, Steiner B, Brandt M-D, Jessberger S, Yamaguchi M, et al (2003) Subpopulations of proliferating cells of the adult hippocampus respond differently to physiologic neurogenic stimuli. *Journal of Comparative Neurology* 467(4): 455–463. doi: 10.1002/cne.10945 14624480 10.1002/cne.10945

[68] Tapia-Arancibia L, Aliaga E, Silhol M, Arancibia S (2008) New insights into brain BDNF function in normal aging and Alzheimer disease. *Brain research reviews* 59(1): 201–220. doi: 10.1016/j.brainresrev.2008.07.007 18708092 10.1016/j.brainresrev.2008.07.007

[69] Smith S-M, Jenkinson M, Johansen-Berg H, Rueckert D, Nichols T-E, Mackay C-E, et al (2006) Tract-based spatial statistics: voxelwise analysis of multi-subject diffusion data. *Neuroimage* 31(4): 1487–1505. doi: 10.1016/j.neuroimage.2006.02.024 16624579 10.1016/j.neuroimage.2006.02.024

