# Supplementary material for: Dance training is superior to repetitive physical exercise in inducing brain plasticity in the elderly
Source: PLoS One. 2018 Jul 11;13(7):e0196636. doi: 10.1371/journal.pone.0196636 (PMC6040685; doi:10.1371/journal.pone.0196636)
Supplement: S1 Table — Annotation. BMI = Body-Mass-Index; BDI-II = Becks-Depressions-Inventar II; MMSE = Mini Mental State Examination. (PDF) [file pone.0196636.s001.pdf]

S1 Table. Characteristics of the dance and sport group (M = Mean; SD = Standard deviation).

	**Dance Group n = 20**		**Sport Group n = 18**			
	**M**	**SD**	**M**	**SD**	**T-value**	**p-value**
**Age [Years]**	68.16	4.31	68.72	2.68	.475	.638
**Sex [%]**	40% male		56% male		.945	.351
**BMI [kg/m²]**	26.65	3.54	27.41	3.50	.655	.517
**Education [Years]**	15.32	2.34	16.33	1.40	1.605	.118
**BDI-II [Points]**	5.89	3.26	4.67	3.99	1.028	.311
**MMSE [Points]**	28.32	.946	28.83	.786	1.478	.076

Annotation. BMI = Body-Mass-Index; BDI-II = Becks-Depressions-Inventar II; MMSE = Mini Mental State
