# Supplementary material for: Dance training is superior to repetitive physical exercise in inducing brain plasticity in the elderly
Source: PLoS One. 2018 Jul 11;13(7):e0196636. doi: 10.1371/journal.pone.0196636 (PMC6040685; doi:10.1371/journal.pone.0196636)
Supplement: S2 Table — Annotation. lGTS = left gyrus temporalis superior, lGPre = left gyrus precentralis, MCC = medial cingular cortex, lSMA = left supplementary-motor area, lGPo = left gyrus postcentralis, ACC = anterior cingular cortex, lGFM = left gyrus frontalis medius, rGF = right gyrus fusiformis, lGL = left gyrus lingualis, rTP = right temporalpol, V1 = primary visual cortex, ***p ≤ .001 (uncorrected). (PDF) [file pone.0196636.s002.pdf]

S2 Table. MNI-coordinates and statistical values for gray matter.

**Dance>Sport**	**region**	**t-value**	**z-value**	**p (uncorr.)**	**x (mm)**	**y (mm)**	**z (mm)**
	lGTS	4.53	3.97	0.000***	-57	-39	15
	Insula	4.50	3.95	0.000***	-39	23	3
	lGPre	4.32	3.82	0.000***	-50	-13	12
	MCC	4.26	3.78	0.000***	0	12	34
	lSMA	3.46	3.17	0.001***	-2	11	45
	lGPo	4.23	3.75	0.000***	-53	-24	18
	ACC	4.07	3.64	0.000***	0	38	9
	lGFM	3.78	3.42	0.000***	-38	3	57
**Sport>Dance**	**region**	**t-value**	**z-value**	**p (uncorr.)**	**x(mm)**	**y (mm)**	**z (mm)**
	rGF	4.88	4.20	0.000***	32	-33	-21
	lGL	4.80	4.15	0.000***	-2	-70	4
	rTP	4.52	3.96	0.000***	23	8	-38
	V1	4.15	3.70	0.000***	-8	-97	-8
	Cerebellum	3.98	3.57	0.000***	33	-64	-20

Annotation. lGTS = left gyrus temporalis superior, lGPre = left gyrus precentralis, MCC = medial cingular cortex, lSMA = left supplementary-motor area, lGPo = left gyrus postcentralis, ACC = anterior cingular cortex, lGFM = left gyrus frontalis medius, rGF = right gyrus fusiformis, lGL = left gyrus lingualis, rTP = right temporalpol, V1 = primary visual cortex, ***p ≤ .001 (uncorrected).
