# Supplementary material for: Dance training is superior to repetitive physical exercise in inducing brain plasticity in the elderly
Source: PLoS One. 2018 Jul 11;13(7):e0196636. doi: 10.1371/journal.pone.0196636 (PMC6040685; doi:10.1371/journal.pone.0196636)
Supplement: S3 Table — Annotation. CC-T = truncus of corpus callosum, CC-S = splenium of corpus callosum, AWS = anterior white matter, PWS = posterior white matter, TWS = temporal white matter, OWS = occipital white matter, ***p ≤ .001 (uncorrected). (PDF) [file pone.0196636.s003.pdf]

S3 Table. MNI-coordinates and statistical values for white matter

**Dance>Sport**	**region**	**t-value**	**z-value**	**p (uncorr.)**	**x (mm)**	**y (mm)**	**z (mm)**
	CC-T	4.93	4.24	0.000***	15	6	28
	CC-S	4.47	3.92	0.000***	0	-31	15
	AWS	3.82	3.45	0.000***	-33	-3	46
	PWS	4.22	3.75	0.000***	-36	-36	30
**Sport>Dance**	**region**	**t-value**	**z-value**	**p (uncorr.)**	**x (mm)**	**y (mm)**	**z (mm)**
	TWS	3.95	3.55	0.000***	30	9	-38
	OWS	3.86	3.48	0.000***	21	-82	-2

Annotation. CC-T = truncus of corpus callosum, CC-S = splenium of corpus callosum, AWS = anterior white matter, PWS = posterior white matter, TWS = temporal white matter, OWS = occipital white matter, ***p ≤ .001 (uncorrected).
