# Supplementary material for: Dance training is superior to repetitive physical exercise in inducing brain plasticity in the elderly
Source: PLoS One. 2018 Jul 11;13(7):e0196636. doi: 10.1371/journal.pone.0196636 (PMC6040685; doi:10.1371/journal.pone.0196636)
Supplement: S4 Table — Annotation. Repeated-measures ANOVA following post hoc pairwise comparison (Bonferroni). (PDF) [file pone.0196636.s004.pdf]

S4 Table. BDNF serum and plasma levels before and after intervention.

	Dance Group n = 19				Sport Group n = 16			
	**M**		**SEM**		**M**		**SEM**	**p-value (DG vs SG)**
BDNF Plasma level [pg/ml] pre	1509	±	231		2087	±	316	.142
BDNF Plasma level [pg/ml] post	2220	±	214		2048	±	281	.623
p-value (pre vs post intervention)	.007*							
p-value (interaction effect]				.046*				
BDNF Serum level [pg/ml] pre	35238	±	2269		30442	±	2637	.175
BDNF Serum level [pg/ml] post	36348	±	2403		29724	±	2758	.078
p-value (pre vs post intervention)	.354							
p-value (interaction effect)				.303				

Annotation. Repeated-measures ANOVA following post hoc pairwise comparison (Bonferroni).
