# Supplementary material for: Dance training is superior to repetitive physical exercise in inducing brain plasticity in the elderly
Source: PLoS One. 2018 Jul 11;13(7):e0196636. doi: 10.1371/journal.pone.0196636 (PMC6040685; doi:10.1371/journal.pone.0196636)
Supplement: S5 Table — Annotation. Testbattery for Attention: Alter_vis = alertness (visual), Alert_aud = alertness (+ auditive distractor), Go/NoGo = selective attention (RT = reaction time; F = false; M = missed), D_Att_vis = devided attention (visual), Dev_Att_aud = devided attention (auditive), Flex = flexibility; ZVT = Zahlenverbindungstest (trail making; processing speed), level of significance: p < .05. (PDF) [file pone.0196636.s005.pdf]

S5 Table. Mean and standard deviation of performances in the domain Attention in both groups.

	**Dance Group**				**Sport Group**				**ANOVA**					
	Pre		Post		Pre		Post		Group		Time		Group x Time	
	M	SD	M	SD	M	SD	M	SD	F	p	F	p	F	P
Alert_vis [RT]	293	72	277	42	263	33	255	42	3.63	.064	2.98	.092	0.42	.522
Alert_aud [RT]	284	66	259	27	255	27	245	28	3.76	.059	9.04	.005	1.51	.226
Go/NoGo [RT]	428	56	441	58	424	55	423	65	0.44	.511	0.69	.410	0.96	.333
Go/NoGo [F]	1.52	1.4	4.04	1.0	1.50	1.4	1.25	1.3	0.11	.747	1.92	.174	0.19	.667
Go/NoGo [M]	0.61	1.5	0.17	0.6	0.20	0.5	0.20	0.5	0.91	.346	1.23	.275	1.23	.275
D_Att_vis [RT]	917	130	910	88	847	70	842	86	6.51	.015	0.24	.624	0.00	.969
D_Att_aud [RT]	651	86	637	93	652	114	661	146	0.16	.690	0.03	.864	0.80	.375
D_Att [F]	2.04	1.7	1.35	1.9	1.15	0.9	1.30	1.7	1.19	.280	1.34	.254	3.22	.080
D_Att [M]	2.09	2.7	2.39	2.2	1.25	1.4	1.30	1.1	3.64	.064	0.27	.608	0.14	.712
Flex [RT]	971	243	892	249	864	223	839	237	1.86	.181	1.47	.232	0.37	.546
Flex [F]	3.52	6.9	2.09	2.9	1.85	2.3	1.25	2.1	1.73	.195	1.45	.236	0.24	.624
ZVT [bit/sec.]	32.2	7.1	34.3	7.7	36.6	6.9	37.5	8.5	2.25	.141	1.98	.167	0.40	.851

Annotation. Testbattery for Attention: Alter_vis = alertness (visual), Alert_aud = alertness (+ auditive distractor), Go/NoGo = selective attention (RT = reaction time; F = false; M = missed), D_Att_vis = devided attention (visual), Dev_Att_aud = devided attention (auditive), Flex = flexibility; ZVT = Zahlenverbindungstest (trail making; processing speed), level of significance: $p < .05$.
