# Supplementary material for: Dance training is superior to repetitive physical exercise in inducing brain plasticity in the elderly
Source: PLoS One. 2018 Jul 11;13(7):e0196636. doi: 10.1371/journal.pone.0196636 (PMC6040685; doi:10.1371/journal.pone.0196636)
Supplement: S6 Table — Annotation. VLMT = Verbaler Lern-und Merkfähigkeitstest (verbal learning and memory test): VLMT-L = Learning, VMLT_RI = recall after interference list, VLMT_DR = delayed recall, VLMT_REC = recognition; ROCFT = Rey-Osterrieth Complex Figure Test, ROCFT_C = copy, ROCFT_IR = immeadiate recall, ROCFT_DR = delayed recall, ROCFT_REC = recognition; WMS_DS_fw = Wechselr-Memory Scale (Digit Span forward); pt = points. (PDF) [file pone.0196636.s006.pdf]

S6 Table. Mean and standard deviation of performances in the domain Memory in both groups.

	Dance Group				Sport Group				ANOVA					
	Pre		Post		Pre		Post		Group		Time		Group x Time	
	M	SD	M	SD	M	SD	M	SD	F	P	F	P	F	p
VLMT_L [pt]	47.4	8.8	46.3	7.7	48.9	8.8	50.43	9.0	1.52	.224	0.03	.858	1.16	.287
VLMT_RI [pt]	10.0	3.2	9.04	3.0	10.3	3.0	10.38	3.0	1.01	.322	1.09	.303	1.65	.206
VLMT_DR [pt]	10.0	2.8	9.1	3.1	10.0	3.2	10.43	3.2	0.63	.432	0.26	.615	2.55	.188
VLMT_REC [pt]	11.4	3.5	10.3	3.8	11.1	4.1	11.29	3.0	0.13	.719	0.63	.431	1.49	.229
ROCFT_C [pt]	30.2	4.6	31.7	4.5	32.8	2.7	33.	1.4	3.99	.052	2.86	.099	1.48	231
ROCFT_IR [pt]	16.1	6.0	21.5	7.9	18.5	5.5	24.38	5.6	2.40	.129	39.47	.000	0.05	.827
ROCFT_DR [pt]	16.1	5.3	20.8	7.5	18.7	5.5	24.24	6.1	3.47	.070	31.52	.000	0.22	.646
ROCFT_REC [pt]	20.5	1.5	19.3	2.7	19.0	1.5	18.55	2.1	4.70	.037	4.30	.045	0.61	.442
WMS_DS_fw [pt]	7.7	1.8	8.1	2.1	8.3	1.8	8.44	1.7	0.78	.383	0.61	.439	0.04	.841

Annotation. VLMT = Verbaler Lern-und Merkfähigkeitstest (verbal learning and memory test): VLMT-L = Learning, VMLT_RI = recall after interference list, VLMT_DR = delayed recall, VLMT_REC = recognition; ROCFT = Rey-Osterrieth Complex Figure Test, ROCFT_C = copy, ROCFT_IR = immeadiate recall, ROCFT_DR = delayed recall, ROCFT_REC = recognition; WMS_DS_fw = Wechselr-Memory Scale (Digit Span forward); pt = points
