# Supplementary material for: Dance training is superior to repetitive physical exercise in inducing brain plasticity in the elderly
Source: PLoS One. 2018 Jul 11;13(7):e0196636. doi: 10.1371/journal.pone.0196636 (PMC6040685; doi:10.1371/journal.pone.0196636)
Supplement: S7 Table — Annotation. WMS_DS_bw = Wechsler Memory Scale (Digit Span backward); RWT = Regensburger Wortflüssigkeitstest (verbal word fluency), RWT_M-Words = formallexikal word fluency, RWT_G-R-words = formallexikal. shift between categories, RWT_animals = semantic word fluency, RWT_Cl-Fl = Clothing and flowers (semantic shift of category). (PDF) [file pone.0196636.s007.pdf]

S7 Table. Mean and standard deviation of performances in the domain Executive Functions in both groups.

	**Dance Group**				**Sport Group**				**ANOVA**					
	**Pre**		**Post**		**Pre**		**Post**		**Group**		**Time**		**Group x Time**	
	**M**	**SD**	**M**	**SD**	**M**	**SD**	**M**	**SD**	**F**	**P**	**F**	**P**	**F**	**P**
WMS_DS_bw [pt]	6.05	1.5	6.2	1.6	6.8	1.7	6.8	1.7	2.25	.142	0.09	.763	0.09	.763
RWT_M-Words [pt]	17.7	5.8	17.8	4.9	18.1	3.8	19.0	4.9	0.42	.520	0.35	.560	0.24	.631
RWT_G-R-Words [pt]	19.5	4.3	19.0	4.3	21.5	4.9	21.8	5.6	3.57	.066	0.02	.902	0.21	.649
RWT_animals [pt]	30.7	7.1	30.5	6.2	32.9	5.1	33.3	7.3	2.23	.143	0.00	.971	0.05	.818
RWT_Cl-Fl [pt]	19.7	4.3	21.0	5.4	20.4	4.0	20.5	4.5	0.00	.963	1.26	.269	0.92	.342

Annotation. WMS_DS_bw = Wechsler Memory Scale (Digit Span backward); RWT = Regensburger Wortflüssigkeitstest (verbal word fluency), RWT_M-Words = formallexikal word fluency, RWT_G-R-words = formallexikal. shift between categories, RWT_animals = semantic word fluency, RWT_Cl-Fl = Clothing and flowers (semantic shift of category).
