# Supplementary material for: Dance training is superior to repetitive physical exercise in inducing brain plasticity in the elderly
Source: PLoS One. 2018 Jul 11;13(7):e0196636. doi: 10.1371/journal.pone.0196636 (PMC6040685; doi:10.1371/journal.pone.0196636)
Supplement: S2 File — (PDF) [file pone.0196636.s009.pdf]

Deutsches Register Klinischer Studien
German Clinical Trials Register

PLEASE NOTE: *This trial has been registered retrospectively.*

Trial Description

Title

The impact of dancing versus health-related physical exercise on structural neuroplasticity, working memory and selected cognitive and motor functions in senior citizen.

Trial Acronym

[---]*

URL of the trial

[---]*

Brief Summary in Lay Language

Aging goes along with cognitive and motor (physical) decline. Attention, memory and perceptual processes decreases. Strength and endurance ability get reduced and balance deteriorates. The orientation of the body in space as well as reaction abilitiy is also characterized by decreased performances in the elderly. But, the trainabilty and especially the neuroplasticity remains! Several studies indicated that endurance training, strength training as well as motor skill training counteract aging processes. But so far, the impact of dancing, which set demands on conditional, coordinative and cognitive abilities compared to endurance or strength training has not been investigated jet. So the aim of this study was to examine the impact of a six months lasting dance training compared to a endurance and strength training on the brain structure, cognitive functions and motor abilities in seniors. Our finding could make a contribution to recommendation for senior sport.

Brief Summary in Scientific Language

Two exercise programs have been compared:
1. Dance training, which is characterized by acyclic movement structures and permanent learning process of arm- and leg patterns to changing genres (Latin American, Jazz Dance, Square Dance, Line Dance).
2. Endurance and strength-endurance, which is composed of cyclic, alternate structures. Endurance training was performed on a bicycle ergometer according to the individual calculated training heart frequency. Endurance-strength training was executed with and without equipment (barbells, rubberbands). The combination of body segments was avoided.

Dancing features multimodal sensorimotor stimuli for instance: The interconnection of arm and leg patterns to music, the position of the body and body segments in space and to the fellow dancers, the permanent change of single leg stances, turns and shift in the center of gravity, memory and recall of arm-and leg pattern to certain parts of the music under the pressure of time, as well the permanent learning process of movement sequences under conditional

load leads to the assumption, that a six-months lasting dance training indicate stronger structural brain changes, as well as cognitive and motor performance alterations compared to a monotonous, cyclic endurcance and strenght-endurance training.

Organizational Data

- DRKS-ID: **DRKS00012605**
- Date of Registration in DRKS: **2017/07/24**
- Date of Registration in Partner Registry or other Primary Registry: **[---]***
- Investigator Sponsored/Initiated Trial (IST/IIT): **yes**
- Ethics Approval/Approval of the Ethics Committee: **Approved**
- (leading) Ethics Committee Nr.: **22/12 , Ethikkommission der Medizinischen Fakultät der Otto-von-Guericke-Universität Magdeburg**

Secondary IDs

Health condition or Problem studied

- Free text: **healthy elderly**

Interventions/Observational Groups

- Arm 1: **Dance group,practiced twice a week, 90 minutes each unit**
  - changing formation and choreographies evry 4th unit (Line Dance, Jazz Dance, Square Dance, Latin American Dance)
  -accompanied by music.

  The intervention lasted for 6 months.
- Arm 2: **Sport/fitness group, twice a week for 90 minutes each unit**
  -accompanied by music.
  The intervention lasted 6 months.

Characteristics

- Study Type: **Interventional**
- Study Type Non-Interventional: **[---]***
- Allocation: **Randomized controlled trial**
- Blinding: **[---]***

Study Type: **Interventional**

Study Type Non-Interventional: **[---]***

Allocation: **Randomized controlled trial**

Blinding: **[---]***

- Who is blinded: **[---]***
- Control: **Active control (effective treament of control group)**
- Purpose: **Prevention**
- Assignment: **Parallel**
- Phase: **N/A**
- Off-label use (Zulassungsüberschreitende Anwendung eines Arzneimittels): **N/A**

Primary Outcome

1. Structural changes in gray and white matter volume:

Structural data were assessed using 3T MRI and analysed using VBM (voxel based morphometry) (SPM12) and DTI (Diffusions-Tensor Imaging).

2. Investigation in changes of BDNF-Level (Brain-Derived-Neurotrophic Factor):

Blood samples were taken under fast conditions and analysed regarding the neurotrophin BDNF.

3. Cognitive Testing:
Testbattery to assess Attention (TAP), verbal learn and memory test (VLMT), Rey-Osterrieth-Complex-Figure Test (ROCFT), Mini Mental Status Examination (MMSE), Verbal word fluency test, Wechsler Memory Scale-R (Number Span forward and backward), Trail-Making Test (German Version:ZVT), CANTAB

3. Physical Testing:
motor sequence leraning test, Physical Working Capacity 130, Spatial Navigation Test, Balnace Test (Sensory Organisation Test and Limits of Stability)

Basline: June 2012 - September 2012
Post-test: April 2013 - June 2013

Secondary Outcome

Questionnaire for Biography of Physical Activity (Wollny, 2002) throughout the life span (childhood, youth, young, middle and late adulthood)

Screeings-Assessments to exclude/include subjects:
- Mini Mental Status Examination (Folstein et al., 1990) to assess cognitive health
- Becks-Depression-Iventory II
- Freiburger Questionnaire for Physical Activity
- Heath Questionnaire to assess neurologic or other diseases, medical treatments, physical activity.

During the intervention we used a questionnaire to assess daily activity and the sleep-behavior (IPAQ - International Physical Activity Questionnaire)

Countries of recruitment

- DE **Germany**

Locations of Recruitment

- other **Deutsches Zentrum für Neurodegenerative Erkrankungen, Magdeburg**

Recruitment

- Planned/Actual: **Actual**
- (Anticipated or Actual) Date of First Enrollment: **2012/04/04**
- Target Sample Size: **60**
- Monocenter/Multicenter trial: **Monocenter trial**
- National/International: **National**

Inclusion Criteria

- Gender: **Both, male and female**
- Minimum Age: **65 Years**
- Maximum Age: **80 Years**

Additional Inclusion Criteria

mental health (MMSE > 27 points), no physical or health issues

Exclusion criteria

Metalic implants, neurologic diseases, regular physical training (>1/week), tattoos, claustophobia, tinnitus, depressive symptoms (BDI-II)

Addresses

- **Primary Sponsor**

  Deutsches Zentrum für Neurodegenerative Erkrankungen (Helmholtz Gemeinschaft)

Primary Sponsor

Deutsches Zentrum für Neurodegenerative Erkrankungen (Helmholtz Gemeinschaft)
Sigmund-Freud-Straße 27
53127 Bonn
Germany

Telephone: **[---]***
Fax: **[---]***
E-mail: **[---]***
URL: **www.dzne.de**

- **Contact for Scientific Queries**

Deutsches Zentrum für Neurodegenerative Erkrankungen
Prof. Dr. Notger Müller
Leipziger Str. 44/Haus 64/5.Etage
39120 Magdeburg
Germany

Telephone: **0391-6724517**
Fax: **[---]***
E-mail: **notger.mueller at dzne.de**
URL: **www.dzne.de**

- **Contact for Public Queries**

Otto-von-Guericke Universität Institut III - Bereich Sportwissenschaft
Dr. Kathrin Rehfeld
Zschokkestaße 32/Geb. 40-D/R075
39104 Magdeburg
Germany

Telephone: **0391-6754706**
Fax: **[---]***
E-mail: **kathrin.rehfeld at ovgu.de**
URL: **www.ispw.ovgu.de**

Sources of Monetary or Material Support

- **Institutional budget, no external funding (budget of sponsor/PI)**

Deutsches Zentrum für Neurodegenerative Erkrankungen
Sigmund-Freud-Straße 27
53127 Bonn
Germany

Telephone: **[---]***

Institutional budget, no external funding (budget of sponsor/PI)

Deutsches Zentrum für Neurodegenerative Erkrankungen
Sigmund-Freud-Straße 27
53127 Bonn
Germany

Telephone: **[---]***
Fax: **[---]***
E-mail: **[---]***
URL: **www.dzne.de**

- **Institutional budget, no external funding (budget of sponsor/PI)**

Otto-von-Guericke Universität Magdeburg
Universitätsplatz 2
39106 Magdeburg
Germany

Telephone: **[---]***
Fax: **[---]***
E-mail: **[---]***
URL: **www.ovgu.de**

Status

- Recruitment Status: **Recruiting complete, follow-up complete**
- Study Closing (LPLV): **2013/09/30**

Trial Publications, Results and other documents

- Paper **Dancing or Fitness Sport? The Effects of Two Training Programs on Hippocampal Plasticity and Balance Abilities in Healthy Seniors**

** This entry means the parameter is not applicable or has not been set.*
**** This entry means that data is not displayed due to insufficient data privacy clearing.*
