# Supplementary material for: Dance training is superior to repetitive physical exercise in inducing brain plasticity in the elderly
Source: PLoS One. 2018 Jul 11;13(7):e0196636. doi: 10.1371/journal.pone.0196636 (PMC6040685; doi:10.1371/journal.pone.0196636)
Supplement: S3 File — (DOCX) [file pone.0196636.s010.docx]

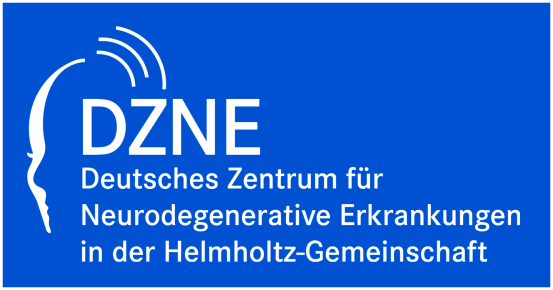

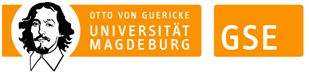


Antrag auf Genehmigung eines Forschungsvorhabens durch die Ethikkommission

Antragsteller:

Frau Prof. Dr. A. Hökelmann, Institut für Sportwissenschaft

Herr Prof. Dr. med. N. Müller, DZNE & Universitätsklinik für Neurologie

Frau Kathrin Rehfeld, Institut für Sportwissenschaft

**Thema des Forschungsvorhabens**

Untersuchungen zum Einfluss eines Tanz- und eines gesundheitssportlichen Trainings auf die Neurogenese im Gehirn, auf das Arbeitsgedächtnis und auf ausgewählte kognitive und motorische Funktionen bei Senioren.

- Untersuchung zu Auswirkungen eines Tanz- und gesundheitssportlichen Trainings bei älteren gesunden Probanden auf:

1. *Die hippocampale Neurogenese gemessen mittels Magnetresonanztomographie (MRT), und funktioneller MRT*
2. *Die Produktion von Hirnwachstumsfaktoren (BDNF)*

- Untersuchung zu Auswirkungen eines Tanz- und gesundheitssportlichen Trainings auf hippocampusspezifische kognitive Leistungen (Mustererkennung)
- Untersuchung der Abhängigkeit des Trainingserfolgs von genetischen Faktoren
- Untersuchung zu Auswirkungen eines Tanz- und gesundheitssportlichen Trainings bei älteren gesunden Probanden auf die Informationsverarbeitungsgeschwindigkeit, Konzentration, Aufmerksamkeit, Gedächtnisleistungen (siehe neuropsychologische Tests)
- Untersuchung zu Auswirkungen eines Tanz- und gesundheitssportlichen Trainings bei älteren gesunden Probanden auf:

1. *die motorische Lernfähigkeit, bestimmt durch quantitative Bewegungsparameter; gemessen durch eine 3D- Bewegungsanalyse (Vicon-System).*
2. *das statische Gleichgewicht, das dynamische Gleichgewicht und auf die Reaktionszeit (Balance-Master „Smart Equitest System“)*
3. *Anpassungen des Belastungs- und Erholungspulses, erfasst durch einen Belastungs-Beanspruchungstest (Steptest) sowie dem PWC 130*

**Hintergrund des Forschungsvorhabens**

Die demographische Entwicklung stellt die Gesellschaft im 21. Jahrhundert vor besondere Herausforderungen bezüglich der Demenzprävention. Infolge einer immer älter werdenden Bevölkerung und der damit verbundenen exponentiell ansteigenden Demenz-Prävalenz ist es zwingend notwendig, wirkungsvolle Trainingsangebote zum Erhalt der körperlichen und geistigen Funktionen im Alter zu erarbeiten und zu testen. Dadurch soll es möglich werden, eine hohe Lebensqualität (Selbstständigkeit) lange zu erhalten und den steigenden finanziellen Belastungen im Gesundheitssektor entgegenzuwirken. Alter und Altern gehen mit einer Reihe physiologischer, neurophysiologischer und kognitiver Veränderungen einher. So nimmt im Altersgang die Muskelmasse um 40% ab und beeinflusst die konditionelle und koordinative Leistungsfähigkeit negativ (Baumann, 1996).

Im Gehirn nimmt die Durchblutung ab, es lagern sich Stoffwechselendprodukte ein (ß-Amyloid-Plaque), die Anzahl der Nervenzellen nimmt ab (Voelcker-Rehage, Godde & Staudinger, 2006) und der Hippocampus atrophiert (Small et al., 2002). Reduzierungen in physiologischen und neurophysiologischen Mechanismen beeinflussen nachhaltig die kognitive Leistungsfähigkeit.

Nicht alle Alterserscheinungen sind genetisch determiniert oder irreversibel, vielmehr können einige Erscheinungen teilweise verzögert oder vermieden werden.

Verschiedene Trainingsstudien zeigen, dass durch gezielte körperliche Aktivität, bzw. durch ein kontinuierliches Training dem Leistungsabfall entgegengewirkt werden kann (u.a. Conzelmann, 1993; Teipel & Kemper, 1997; Freiberger, 2001). Die durchweg besseren Befunde trainierender Personen im Vergleich zu gleichaltrigen nicht trainierenden lassen darauf schließen, dass die Bewegungskoordination bei älteren Menschen in Folge regelmäßigen Übens positiv beeinflussbar ist. So zeigen Interventionsstudien zum Erhalt der Gleichgewichtsfähigkeit (Hu et al., 1994; Lord et al., 1996; LI etal., 2007; Cyatro et al., 2008), wie effektiv ein Training für deren Erhalt bzw. Verbesserung sein kann. LI et.al. (2007) konnten nach einer 12-monatigen Tai-Chi-Intervention eine signifikante Verbesserung der Balancefähigkeit (ø 71,8 Jahre) nachweisen. Zu ähnlichen Ergebnissen kommt die Studie von Cyatro et al. (2008). Bereits nach einem 20-wöchigen Haus- und Gruppentraining verbesserte sich die Gleichgewichtsfähigkeit bei 65-96 jährigen Menschen erheblich. Diese Studien zeigen, dass auch Menschen im höheren Alter motorisch vielfältig trainierbar sind.

Die Verbesserung der Bewegungskoordination, speziell die Stabilisierung der Gleichgewichtsfähigkeit, ist auf die Plastizität des Organismus zurückzuführen, da viele Funktionen im kognitiven und motorischen Bereich lebenslang plastisch, d.h. form- und veränderbar sind. Diese Formbarkeit lässt sich auch bei neurophysiologischen Parametern beobachten, die durch körperliche Aktivität positiv beeinflussbar sind. Es ist bekannt, dass im späteren Erwachsenenalter eine hippocampale Atrophie eintritt (Small et al., 2002). Das Ausmaß des Gewebsuntergangs kann aber durch verschiedene Faktoren wie z.B. körperliche Aktivität und kognitive Stimulation verlangsamt werden (Flöel, 2008), da Neurogenese (Nervenneubildung) auch im späteren Erwachsenalter erfolgen kann.

Derzeit überwiegen vor allem Studien zum Einfluss von Ausdauerbelastungen auf kognitive und mentale Prozesse. Es konnte nachgewiesen werden, dass sich ein Ausdauertraining positiv auf die kognitiven Leistungen auswirkt (Hillman et al., 2008) und eine Neurogenese im Gyrus dentatus (Bereich des Hippocampus) stattfindet.

Im Tierversuch konnte durch ein Laufradtraining mit älteren Mäusen (18 Monate) eine Zunahme von Nervenzellen im Gyrus dentatus sowie verbesserte Leistungen in einer „Pattern Separation Task“ beobachtet werden (Praag et al. 2005). Vergleichbare Humanstudien stehen derzeit noch aus. Jedoch konnte auch im älteren menschlichen Gehirn Plastizität bewiesen werden (Boyke et al., 2008). Erickson et al. (2009) unterzogen älteren Probanden im durchschnittlichen Alter von 66,5 Jahren einem standardisierten Fitnesstest und fanden Korrelationen zwischen körperlicher Fitness, Hippocampusgröße und visuell-räumlichen Gedächtnisleistungen. Nach Ausdauerinterventionen wird über verbesserte kognitive Leistungen im Bereich der Konzentrationsfähigkeit, des Arbeitsgedächtnisses, des Setzens von Zielen sowie des Planungsvermögens berichtet (Hillman, Erickson & Kramer, 2008). Voelcker-Rehage, Godde und Staudinger (2008) verdeutlichen, dass nicht nur regelmäßiger Ausdauersport die kognitive Leistungsfähigkeit steigert, sondern auch Gymnastik. In der Meta-Analyse von Colcombe und Kramer (2003) konnte bewiesen werden, dass sich ausdauer- und kraftinduziertes Training positiv auf kognitive Leistungen auswirkt. Dabei wurden stärkere Zusammenhänge hinsichtlich höherer Denkprozesse (Koordination, Planung, Arbeitsgedächtnis) gefunden. Eine Vielzahl von Studien belegt, dass durch sportliche Betätigung eine Volumenzunahme des Gehirns zu verzeichnen ist und dem Abbauprozess des Gehirns, beginnend mit dem dritten Jahrzehnt, entgegen gewirkt (Erickson, Prakash, Voss et al. 2009) werden kann.

Neuste Forschungsergebnisse verdeutlichen, dass Tanzen im Vergleich zum Gesundheitssport/Fitnesssport (d.h. ausdauer- und kraftinduziertes Training) signifikante Verbesserungen im Bereich der fluiden Intelligenz (Informationsverarbeitungsgeschwindigkeit), der allgemeinen Intelligenz (Raumvorstellung, Sprachverständnis, Wahrnehmungsdifferenzierung, schlussfolgerndes Denken) und dem Arbeitsgedächtnis bei Senioren im Alter zwischen 60 bis 75 Jahren hervorruft (Hökelmann, Lehmann, Rehfeld & Blaser, 2011). Offensichtlich wird durch das Tanzen, speziell durch die simultane und sukzessive Bewegungskopplung, durch das Abrufen komplexer Schritt- und Armmuster unter dynamischen, rhythmischen und konditionellen Druckbedingungen Reize gesetzt, die sich stärker auswirken, als vergleichsweise zyklische und automatisierte Bewegungen, wie Laufen oder Fahrradfahren.

**Ziele des Forschungsvorhabens**

Tanzen bietet im Vergleich zu Sportarten, die auf zyklischen Bewegungsmustern basieren, vielfältige Potenzen konditionelle, koordinative und kognitiven Fähigkeiten zu trainieren und fördert außerdem eine soziale Integration.

Anhand einer randomisierten Studie soll untersucht werden, inwieweit ein mehrmonatiges Tanztraining im Vergleich zu einem gesundheitssportorientierten Gruppentraining, Auswirkungen auf den neurophysiologischen, kognitiven, konditionellen und koordinativen Status bei gesunden Senioren hat. Die Ergebnisse dieser Studie, die primär auf Demenz-Primärprävention und den Erhalt bzw. der Verbesserung körperlicher Funktionen zielt, bilden eine wichtige Grundlage für weiterführende Studien in der Sekundär- und Tertiärprävention.

**Methodischer Aufbau**

Das Forschungsvorhaben ist in eine Prätrainings-, Trainings- und Posttrainingsphase gegliedert. Während der Prä- und Posttrainingsphasen erfolgen Leistungs- und Verhaltensmessungen sowie MRT-, und fMRT-Messungen. Zusätzlich wird den Probanden Blut zur Einschätzung des Gesundheits- und Fitnesszustandes sowie zur Bestimmung molekularbiologischer Werte abgenommen.

**Voruntersuchungen vor Aufnahme der körperlichen Belastung:**

Um die Eignung zur Teilnahme an den Trainingsmaßnahmen zu überprüfen werden Voruntersuchungen durchgeführt. Besteht Zweifel an der Eignung, müssen die Probanden ein ärztliches Attest vorlegen. Folgende Voruntersuchungen erfolgen:

- Gesundheitsfragebogen (Frage nach Vorerkrankungen, Medikamenteneinnahme)
- Blutdruck-Messung
- Bestimmung der Ruhe-Herzfrequenz
- Laborchemische Untersuchungen: Lipidstatus, Blutzucker (HbA1c), kleines Blutbild, TSH-Wert

Untersuchungen nach Durchführung des körperlichen Trainings:

- Bestimmung der Ruhe-Herzfrequenz
- Blutdruck-Messung
- Laborchemische Untersuchungen: Lipidstatus, Blutzucker (HbA1c), kleines Blutbild, TSH-Wert

**Betreuung der einzelnen Sektoren:**

Die sportwissenschaftliche Betreuung, die Durchführung der Interventionsprogramme sowie die Leistungsdiagnostik zur Studie werden von Kathrin Rehfeld (Sportwissenschaftlerin M.A.), Uwe Sobieray (Student im 10. Semester, Studiengang „Sport und Technik“) und weiteren Studenten unter der Leitung von Prof. Dr. Hökelmann (Institut für Sportwissenschaft) übernommen.

Die Analyse der Konzentration des BDNF-Proteins im Blut erfolgt durch das Institut für Physiologie der Universitätsklinik Magdeburg.

Die Bestimmung der genetischen Polymorphismen wird durch das Leibnitz-Institut für Neurobiologie Magdeburg durchgeführt.

Die Verhaltensmessungen werden durch das Deutsche Zentrum für Neurodegenerative Erkrankungen durchgeführt.

**Sportmotorische Untersuchungen**

Alle Probanden absolvieren einen dynamischen und statischen Gleichgewichtstest, wobei auch die Reaktionszeit ermittelt wird. Die Probanden stehen barfuß auf einer Kraftmessplatte und folgen den Anweisungen über einen Bildschirm. Bei der Ausübung verschiedener Gleichgewichtsübungen sind die Probanden in einem Sicherheitsgeschirr eingespannt, um Stürze zu vermeiden.

Zur Ermittlung des Belastungs- und Erholungspulses wird der Belastungs- Beanspruchungstest mit dem Stepbrett durchgeführt. Bei diesem Test wird die Geschwindigkeit durch ein Metronom (116 bpm) vorgegeben. Die Belastungsphase beträgt 2 Minuten, wobei keine maximale Ausbelastung erreicht wird. Für die Erholungsphase wird eine Zeit von 90 Sekunden vorgesehen. Insgesamt besteht der Test aus 3 Belastungs- und 3 Erholungsphasen. Explizit soll ermittelt werden, wie schnell sich die Probanden von der Belastung erholen und zum Ruhepuls zurückkehren. Die Testzeit beträgt 9 Minuten.

Ein weiterer Test zur Erfassung und Entwicklung der körperlichen Leistungsfähigkeit bei Menschen über 50 Jahren, bildet der PWC 130 (physical working capacity). Die Probanden sitzen auf einem Fahrradergometer beginnen zunächst bei einer Belastung von 25 Watt (Schwächere 15 Watt) zu treten. Die Belastung wird alle 2 Minuten um 25 Watt gesteigert bis die Zielherzfrequenz von 130 erreicht wird. Zuzüglich zum Puls wird auch der Blutdruck gemessen. Nach Erreichung des Zielpulses werden nach einer, drei und fünf Minuten der Erholungspuls sowie Blutdruck dokumentiert.

Der motorische Lerntest zielt auf die Erfassung der Qualität der Körperbewegungen nach einem definierten Lernzeitraum. Die Probanden werden auf der Grundlage des Plug and Gait-Modells an spezifischen Gelenkpunkten gemarkert. 12 Vicon-Kameras erfassen ausschließlich die Marker der Probanden. Durch konstitutionelle Eingaben wie Körpergröße, Gewicht, Beinlänge u.a., erstellt das Programm ein Ganzkörpermodell der Probanden. In Echtzeit können Bewegungen ausgeführt und aufgenommen werden (Markerpunkte). Die Probanden müssen simultan einen Armkreis vor dem Körper entgegen der Bewegungsrichtung mit einem Kreuzschritt ausführen (Kopplung zweier Körperzentren mit unterschiedlicher Bewegungsrichtung). Anschließend wird eine Drehung um 180° mit Verlagerung des Körperschwerpunktes und einem abschließenden Armkreis mit Kreuzschritt ausgeführt. An Parametern, wie Weg, Zeit, Winkeln, Geschwindigkeiten, speziell des Bewegungsflusses soll beurteilt werden, inwieweit sich die Qualität der Körperbewegung nach der Intervention verändert hat (Dauer: ca. 30 Minuten; Aufnahmedauer: 30 Sekunden).

In Verbindung mit dem motorischen Lerntest werden die Probanden eine Ganganalyse nach den identischen organisatorischen Prinzipien (Vicon 3D Bewegungsanalyse) absolvieren. Dabei gehen die Probanden entsprechend einer Richtgeschwindigkeit auf dem Laufband und müssen eine Aufgabe lösen (dual task). Zur Sicherheit der Probanden, werden diese wie bei dem Gleichgewichtstest in ein Sicherheitsgeschirr eingespannt, um mögliche Stürze zu vermeiden. Die Dauer der Testung beträgt 3 x 30 Sekunden.

**Verhaltensmessung**

Mit allen Probanden wird eine orientierende neuropsychologische Testung durchgeführt. Die einzelnen Tests sind entweder am Computer oder auf dem Papier zu bearbeiten. In aller Kürze werden die neuropsychologischen Tests aufgeführt:

- Mini Mental Status Examination
- Wechsler Memory Scale-R (Zahlen nachsprechen)
- CANTAB (delayed matching to sample; paired associates learning; spatial span)
- Regensburger Wortflüssigkeitstest
- Rey Osterrieth Complex Figure Test
- Virtuelle Watermaze
- Mehrfach-Wortschatz
- Zahlen-Verbindungs-Test
- Berliner Intelligenzstrukturtest (nur Facette „Merkfähigkeit“)

Zusätzlich werden die Probanden im Rahmen der Erhebung gebeten, psychosoziale Faktoren/Merkmale auf Fragebögen auszufüllen:

- Eysenck-Persönlichkeits Inventar
- NEO-Fünf-Faktoren-Inventar
- Temperament Charakteristic Inventar
- Fragebogen zur Erfassung des Wohlbefindens
- Satisfaction with life scale/ Ryff-Skala
- Life Orientation Test
- Consideration of future consequences scale
- Handlungskontrolle nach Erfolg/Misserfolg
- Becks Depression Inventar-II
- Trierer Inventar zum chron. Stress
- Fragebogen zur sozialen Unterstützung
- Gesundheitsfragebogen zu Lebensgewohnheiten
- Food frequency list
- Freiburger Fragebogen zur körperlichen Aktivität
- Pitsburgh Schlafqualitätsindex
- Oldfield Händigkeitsindex

**MRT-Messung**

**Untersuchungen im 3-Tesla-Kernspintomographen**

Ein Teil der Verhaltensmessung findet für alle Probanden während einer funktionellen MRT-Messung statt. Für diese Messung liegen die Studienteilnehmer auf einem Untersuchungstisch, der in den MR-Tomographen (3 Tesla) langsam hineingefahren wird. Mit einer Signalvorrichtung können sie sich jederzeit dem Untersucher bemerkbar machen. Zudem erfolgt während der gesamten Untersuchungszeit eine kontinuierliche visuelle Überwachung. Nach einem Satz von hochauflösenden Aufnahmen zur späteren Normalisierung der individuellen Daten wird die funktionelle Bildgebung durchgeführt. Insgesamt wird die Untersuchung 50 Minuten dauern.

**Blutuntersuchungen**

In der Prätrainingsphase wird den Probanden Blut zur molekularbiologischen Untersuchung abgenommen. Dieses Blut wird auf einen Hirnwachstumsfaktor (Brain-derived neurotrophic factor- BDNF) sowie auf verschiedene Polymorphismen getestet, von denen vermutet wird, dass sie sich auf die kognitive Leistungsfähigkeit auswirken können. Die Blutabnahme erfolgt durch eine examinierte Krankenschwester oder durch einen Arzt. Die Proben werden im Zentrallabor und am Institut für Neurobiologie analysiert. Die Speicherung der Daten erfolgt in anonymisierter Form in einer Datenbank des DZNE. Dabei wird insbesondere sichergestellt, dass nur unmittelbar an den Untersuchungen beteiligte Personen Zugriff auf die Identität der Versuchsteilnehmer haben.

Zur Einschätzung des allgemeinen Gesundheits- und Fitnesszustandes wird den Probanden zusätzlich Blut in der Prä- und der Posttrainingsphase abgenommen. In diesem Zusammenhang wird auch die Nierenfunktion (Kreatin-Wert) erfasst, die Aufschluss über eventuelle Kontraindikationen einer Kontrastmittelanwendung gibt. Sämtliche Proben- Daten werden spätestens 3 Jahre nach der Blutentnahme vollständig vernichtet.

**Tanztraining**

Probanden die in die Tanzgruppe randomisiert werden, nehmen über einen Zeitraum von 6 Monaten zweimal wöchentlich für 90 Minuten an einem spezifischen Tanztraining (Gruppentraining mit dem Schwerpunkt: Koordinations- und Technikerwerbstraining) in der Sporthalle der Otto- von- Guericke Universität teil.

**Gesundheitssportprogramm**

Probanden dieser Versuchsgruppe nehmen an einem kraft- und ausdauerbasierten Gruppentraining teil, dass auch für 6 Monate zweimal wöchentlich für 90 Minuten in der Sporthalle der Otto- von- Guericke Universität stattfinden wird.

**Probandenauswahl und methodische Risiken**

Insgesamt werden für das Forschungsvorhaben 60 sportlich-inaktive Probanden im Alter von 65 bis 75 Jahren über Zeitungsannoncen rekrutiert. 30 Probanden werden per Zufall in die Tanzgruppe-, 30 Probanden in die Gesundheitssportgruppe eingeteilt. Alle Probanden absolvieren die gleichen Messungen in der Prä-Phase. Nach Beendigung der 6-monatigen Intervention durchlaufen alle Probanden die zweite komplexe Postmessung.

Um in die Studie aufgenommen zu werden, sollten die Probanden in der neuropsychologischen Testung Leistungen zeigen, die der Altersnorm entsprechend als unauffällig einzuschätzen sind. Es werden nur Probanden eingeschlossen, bei denen keine Kontraindikationen gegen Fitnesstraining besteht (normwertiger Blutdruck, BMI < 30, keine relevanten Vorerkrankungen wie Diabetes Mellitus Typ 1, Niereninsuffizienz etc.). Personen, bei denen Kontraindikationen für eine MRT-Untersuchung nicht sicher ausgeschlossen werden können, sind von der Teilnahme an den weiteren Untersuchungen ausgeschlossen. Für die Untersuchung molekularbiologischer und fitnessrelevanter Werte ist die Abnahme von geringen Blutmengen erforderlich. Hierbei bestehen nur die üblichen mit einer Blutabnahme verbundenen Risiken. Das Ergebnis der genetischen Testung ist nach heutigem Wissenstand für die weitere Lebensprognose unerheblich. Die Tatsache, dass die Ergebnisse den Probanden nicht mitgeteilt werden, ist daher ethisch unbedenklich. Alle Ergebnisse der Blutanalysen können von den Probanden eingesehen werden. Bei pathologischen Zufallsbefunden wird ein Arzt hinzugezogen, der über das weitere Vorgehen entscheidet.

**Aufklärung und Einverständniserklärung**

Alle Versuchsteilnehmer/-innen werden ausführlich über die Zielsetzung der Studie und über die verwendeten Methoden aufgeklärt. Insbesondere werden sie auf die anonymisierte Verarbeitung der genetischen Informationen hingewiesen. Sie werden auch darüber informiert, dass sie ihre Bereitschaft zur Teilnahme jederzeit widerrufen können. Die Probanden werden explizit über mögliche Kontraindikationen befragt. Wenn die Probanden mit der Teilnahme an der Studie einverstanden sind und keine Kontraindikationen bestehen, unterschreiben sie die im Anhang beigefügte Einverständniserklärungen.

**Probandenversicherung**

Für die Untersuchungen am DZNE (einschließlich der MR-Messungen) greift das Selbstversicherungsprinzip des Bundes. Eine Unfall- und Wegeversicherung, die darüber hinaus bei solchen Schäden greift, welche nicht der Verantwortung des DZNE zuzurechnen sind, ist derzeit nicht abgeschlossen.

**Literatur**

Baumann, H. (1996). *Fitneß im Alter durch Bewegung*. In: Altersport. Aktuelle Forschungsergebnisse. Denk, H. (Hrsg.). Schorndorf: Verlag Hofmann.

Boyke, J., Driemyer, J., Gaser, C., Buchel, C. & May, A. (2008). Training-induced brain structure changes in the elderly*. J. Neurosci. 28*, 7031-7035

Cyarto, E.V., Brown, W.J., Marshall, A.L. & Trost, S.G. (2008). Comparative Effects of Home- and Group-Based Exercise on Balance Confidence and Balance Ability in Older Adults: Cluster Randomized Trial. Gerontology; 54:272-280

Colcombe, S.J. & Kramer, A.F. (2003). Fitness effects on the cognitive function of older adults: a meta-analytic study. Psychological Science 14: 125–130

Erickson, K.I. & Kramer, A. (2008). Be smart, exercise your heart: Exercise effects on brain and cognition. Nature Reviews Neuroscience (9), Jan. 2008: 58-65.

Erickson, K.I., Prakash, R.S., Voss, M.V., Chaddock, L., Hu, L., Morris, K.S., Whire, S.M., Wojcicki, T.R., McAuley, E. &Kramer, A.F. (2009). Aerobic fitness is associated with hippocampal volume in elderly humans. *HIPPOCAMPUS* 19: 1030- 1039 (2009) Wiley- Liss, Inc.

Flöel, A., Witte, V., Lohmann, H., Wersching, H., Ringelstein, E.B., Berger, K. & Knecht, S. (2008). Lifestyle and memory in the elderly. Neuroepidemiology 2008, 31:39-4.

Freiberger, E. (2001). Zusammenhang von Alter, kognitiven Leistungen und Bewegungskoordination*. Zeitschrift für Gerontopsychologie &-psychiatrie Vol. 14 Nr. 2/2001: 87-100.*

Hillman, C.H., Erickson, K.I. & Kramer, A. (2008). Be smart, exercise ypur heart: exercise effects on brain and cognition. *Nature reviews, Neuroscinece* 9: 58-65.

Hökelmann, A., Lehmann, W., Rehfeld, K. & Blaser, P. (2011). Auswirkungen einer Tanzstudie auf die die kognitive Leistungsfähigkeit älterer Menschen: Eine Magdeburger Tanzstudie. (unveröffentlicht).

Hu, M.H. & Woollacott, M.H. (1994). Multisensory training of standing balance in older adults. J Gerontol 49, M52-71

Jäncke, L. (2009). *Macht Musik schlau? Neue Erkenntnisse aus den Neurowissenschaften und der kognitiven Psychologie*. Bern: Verlag Hans Huber.

Jäger, A.O., Süß, H.-M. & Beauducel, A. (1997). *Berliner Intelligenzstruktur-Test (Form 4).* Göttingen, Bern, Toronto, Seattle: Hogrefe.

[Li, Y](http://www.ncbi.nlm.nih.gov/sites/entrez?Db=pubmed&Cmd=Search&Term=%22Li%20Y%22%5BAuthor%5D&itool=EntrezSystem2.PEntrez.Pubmed.Pubmed_ResultsPanel.Pubmed_DiscoveryPanel.Pubmed_RVAbstractPlus)., [Devault, C.N](http://www.ncbi.nlm.nih.gov/sites/entrez?Db=pubmed&Cmd=Search&Term=%22Devault%20CN%22%5BAuthor%5D&itool=EntrezSystem2.PEntrez.Pubmed.Pubmed_ResultsPanel.Pubmed_DiscoveryPanel.Pubmed_RVAbstractPlus)., [Van Oteghen, S](http://www.ncbi.nlm.nih.gov/sites/entrez?Db=pubmed&Cmd=Search&Term=%22Van%20Oteghen%20S%22%5BAuthor%5D&itool=EntrezSystem2.PEntrez.Pubmed.Pubmed_ResultsPanel.Pubmed_DiscoveryPanel.Pubmed_RVAbstractPlus). (2007). Effects of extended Tai Chi intervention on balance and selected motor functions of the elderly. [Am J Chin Med.](javascript:AL_get(this,%20'jour',%20'Am%20J%20Chin%20Med.'););35(3):383-91.

Lord, S.R., Ward, J.A., Williams, P. (1996). Exercise Effect on Dynamic Stability in Older Women: A Randomized Controlled Trial. Arch of Phys Med and Rehabil 77; 232-36.].

Oswald, W. & Roth, E. (1987). *Der Zahlen-Verbindungs-Test*. Göttingen: Hogrefe.

Small, S.A., Tsai, W.Y., DeLaPaz, R., Mayeux, R. & Stern, Y. (2002). Imaging hippocampal function across the human life span; is memory decline normal or not? Ann Neurol 51:290-295.

Van Praag, H., Shubert, T., Zhao, C. & Gage, F.H. (2005). Exercise enhances learning and hippocampal neurgenesis in aged mice. *The Journal of neuroscience, Sep. 21, 2005 (38): 8680-8685.*

Voelcker-Rehage, C., Godde, B. & Staudinger, U.M. (2006). Bewegung, körperliche und geistige Mobilität im Alter. Bundesgesundheitsblatt- Gesundheitsforschung-Gesundheitsschutz, 49, 558-566, Springer: Wien.
